# The impact of remote care on the quality of care of pregnant women with diabetes: a systematic review and meta-analysis

**DOI:** 10.1016/j.eclinm.2026.104147

**Published:** 2026-08-18

**Authors:** Sara Sousi, Elena Lammila-Escalera, Sevasti Panagiota Glynou, Geva Greenfield, Benedict W.J. Hayhoe, Simon Dryden, Natasha Singh, Azeem Majeed, Ara Darzi, Ana Luísa Neves

**Affiliations:** a Institute of Global Health Innovation, Imperial College London, London, United Kingdom; b Department of Primary Care and Public Health, Imperial College London, London, United Kingdom; c Faculty of Medicine, Barts and the London School of Medicine and Dentistry, London, United Kingdom; d Department of Obstetrics & Gynaecology, Chelsea and Westminster Hospital NHS Foundation Trust, London, United Kingdom; e Department of Metabolism, Digestion and Reproduction, Imperial College London, London, United Kingdom

**Keywords:** Gestational diabetes mellitus, Diabetes in pregnancy, Remote care, Virtual care, mHealth, Quality of care

## Abstract

**Background:**

Diabetes during pregnancy, whether gestational (GDM) or pre-existing, is associated with increased maternal and neonatal risks. Remote care has shown promise in diabetes management, however, its impact on antenatal diabetes care remains unclear. This review evaluates its impact compared with usual care for pregnant women with diabetes, using the Institute of Medicine's healthcare quality framework: effectiveness, safety, efficiency, timeliness, patient-centredness, and equity.

**Methods:**

A systematic review and meta-analysis was conducted. Seven electronic databases (MEDLINE, EMBASE, CINAHL, MIDIRS, Scopus, Cochrane, and Global Health) were searched for studies published between January 01, 2005 and April 30, 2026, with no geographic or language restrictions. Two independent reviewers screened studies and assessed quality using Cochrane tools. A narrative synthesis and meta-analyses were performed. This review was registered with PROSPERO (CRD420251024685).

**Findings:**

Of 1106 unique studies retrieved, 45 were included (n = 26,562), comprising 20 randomised controlled trials (RCTs) and 25 non-randomised studies. Most studies focused on GDM only (n = 37), with the remainder including mixed GDM and pre-gestational diabetes (n = 6) or pre-gestational diabetes only (n = 2). Remote care was generally comparable to usual care across most effectiveness and safety outcomes. In non-randomised studies, remote care was associated with lower unspecified caesarean section rates (relative risk [RR] 0.95, 95% CI 0.91–0.99), postpartum HbA1c (MD −0.08%, 95% CI −0.15 to −0.01), and postpartum 2-h post-prandial glucose levels (MD −1.04 mmol/L, 95% CI −1.59 to −0.48). In RCTs, remote care was associated with reduced emergency caesarean section (RR 0.59, 95% CI 0.38–0.92) and neonatal intensive care unit (NICU) admission (RR 0.73, 95% CI 0.57–0.92). For patient-centredness, patient satisfaction was consistently high. Efficiency findings (cost savings and appointment frequency) were mixed. Three studies assessed timeliness, with no differences observed. Equity outcomes were rarely quantified, though several interventions incorporated cultural, linguistic, or technological adaptations to improve accessibility.

**Interpretation:**

Remote care is a safe and effective alternative to usual care for pregnant women with diabetes, with safety advantages including reduced emergency caesarean sections and NICU admissions. Further research is required to evaluate long-term postpartum outcomes and equitable adoption across diverse populations.

**Funding:**

This study was supported by the 10.13039/501100000272National Institute for Health and Care Research (NIHR) North West London Patient Safety Research Collaboration (NIHR NWL PSRC, NIHR204292).

**Research in context**

**Evidence before this study**

Previous systematic reviews have evaluated telemedicine and virtual care in diabetes management, but few have specifically addressed diabetes in pregnancy or assessed outcomes across all six domains of healthcare quality as defined by the Institute of Medicine, i.e., effectiveness, safety, efficiency, timeliness, equity, and patient-centredness. A 2017 Cochrane review of telemedicine in gestational diabetes reported no significant differences in maternal or neonatal outcomes compared to usual care, while more recent reviews focused primarily on glycaemic control or single outcomes such as patient satisfaction, without a comprehensive synthesis across all maternal and neonatal outcomes. Evidence on cost-effectiveness, equity, and long-term outcomes remains limited and fragmented, with substantial variability in intervention type and study design. We searched MEDLINE, EMBASE, CINAHL, Scopus, Cochrane, MIDIRS, and Global Health databases for studies published between January 01, 2005 and April 30, 2026 using combinations of keywords and MeSH terms, including “remote care”, “telemedicine”, “remote monitoring”, “gestational diabetes”, “pre-gestational diabetes”, and terms reflecting the six domains of healthcare quality (effectiveness, safety, efficiency, timeliness, equity, and patient-centredness). We applied no language restrictions and supplemented our search by screening reference lists and citation searching. Given the increasing prevalence of pregnancies being affected by diabetes, with more than 1 in 7 globally, and the rapid integration of remote care interventions into clinical practice, it is important to investigate the impact of remote care to inform clinical practice, policy, and research.

**Added value of this study**

To our knowledge, this is the first systematic review and meta-analysis to evaluate the impact of remote care for diabetes in pregnancy using the Institute of Medicine's six domains of healthcare quality. By synthesising data from 45 studies involving 26,562 women across multiple countries, this study demonstrates that remote care is largely comparable to usual care for most maternal and neonatal clinical effectiveness and safety outcomes. In non-randomised studies, remote care was associated with lower unspecified caesarean section rates and improved postpartum glycaemic measures, including HbA1c and 2-h post-prandial glucose. In randomised controlled trials (RCTs), remote care was associated with significantly reduced risks of emergency caesarean section (relative risk [RR] 0.59, 95% CI 0.38–0.92) and neonatal intensive care unit (NICU) admission (RR 0.73, 95% CI 0.57–0.92), while patient satisfaction was consistently high across all evaluating studies. This study also incorporates a comprehensive evaluation of efficiency, patient-centredness, and equity, including cultural and linguistic adaptations, which are often underexplored in existing reviews.

**Implications of all the available evidence**

The findings suggest that remote care offers a safe and effective alternative or adjunct to conventional in-person care for pregnant women with diabetes. Although continuous glucose monitoring (CGM) is increasingly utilised in research settings, consensus on targets and management protocols for CGM in gestational diabetes, and particularly in Type 2 diabetes in pregnancy, has not yet been established, and findings from studies incorporating CGM should be considered exploratory. By enhancing accessibility, remote care could alleviate pressure on overstretched maternity services, particularly for women facing travel or logistical barriers. However, gaps remain regarding who benefits the most from remote care, long-term maternal and child health outcomes, cost-effectiveness, and equity, especially in populations with limited digital access or lower digital literacy. Policymakers and healthcare systems should invest in well-designed trials and implementation studies to optimise remote care models, ensure equitable access, and integrate digital health solutions into routine antenatal care pathways.

## Introduction

Diabetes in pregnancy, either gestational diabetes mellitus (GDM) or pre-existing diabetes mellitus (PGDM), is associated with increased maternal and neonatal risks. PGDM refers to type 1 or type 2 diabetes mellitus (DM) diagnosed before pregnancy. GDM affects around 14% of all pregnancies globally, with an increasing prevalence due to risk factors such as obesity and previous pregnancies with GDM.^1^ Key risk factors for GDM include advanced maternal age, elevated body mass index, certain ethnic backgrounds, a history of macrosomic babies, prior GDM, and a family history of diabetes.^2^^,^^3^ Given the rising maternal age and the ongoing obesity epidemic, particularly in Western countries, an increasing number of women are at risk of developing GDM or having a pregnancy affected by diabetes, placing both themselves and their babies at heightened risk of adverse health outcomes.

Pregnant women with PGDM are at an increased risk of hypoglycaemia, diabetic ketoacidosis, worsening diabetic retinopathy and nephropathy, and have higher rates of foetal malformations and miscarriage compared to women without diabetes.^4^ Furthermore, intrauterine exposure to hyperglycaemia increases the risk of type 2 DM (T2DM) in later life for the baby.^5^ All pregnant women with diabetes have an increased risk of pre-eclampsia, premature birth, and caesarean delivery.^6^ Neonatal risks include increased risk of birth defects, hypoglycaemia, macrosomia, and an elevated risk of stillbirth, making closer observation, more frequent patient–clinician interaction, and intensive monitoring central to antenatal care. Also women with GDM are at increased risk of developing obesity and/or T2DM later in life.^7^^,^^8^

Diabetes in pregnancy services are increasingly overburdened by the rising numbers of GDM and PGDM pregnancies which are linked with poorer patient experiences and worsening clinical outcomes.^9^ Furthermore, although screening for GDM is recommended, universal vs risk factor-based screening is often limited by resource capacity rather than risk profiles.^10^ Virtual care platforms and remote monitoring with integration of continuous glucose monitoring have the potential for sustainable, scalable, and patient-centred models that can improve quality of care.

Remote care, including both virtual consultations and remote monitoring, has been increasingly implemented for the management of non-communicable diseases. The Institute of Medicine (IoM) quality of care framework will be used to map the impact on quality of care; this includes patient-centredness, effectiveness, safety, efficiency, timeliness, and equity.^11^ Previous systematic reviews have applied this framework to evaluate remote care in primary care and mental health settings.^12^, ^13^, ^14^, ^15^ A 2025 review involving over 820,000 patients found that virtual consultations are an effective alternative model for glycaemic control (HbA1c) in individuals with T2DM, across the domains of clinical effectiveness, efficiency, patient-centredness, and timeliness. However, mixed results were reported for safety and equity, with key determinants including lower digital literacy and the reduced opportunity for physical assessments.^15^^,^^16^

This systematic review aims to summarise the impact of remote care on the quality of care in the management of diabetes during pregnancy. The specific objectives are: 1) to systematically characterise existing initiatives of remote care in this context; and 2) to summarise the evidence on how these interventions influence quality of care across the six domains of healthcare quality as defined by the IoM.

## Methods

### Search strategy and selection criteria

A systematic literature search was conducted to identify studies examining the impact of remote care on quality of care for pregnant women with diabetes. MEDLINE (via Ovid), EMBASE, Cumulative Index to Nursing and Allied Health Literature (CINAHL), Scopus, Cochrane, Maternity & Infant Care Database (MIDIRS), and Global Health were searched for studies between January 1, 2005 and April 25, 2025. Additional studies were sought by snowballing and reference list checking. The search strategy was developed using the Population, Intervention, Comparison, Outcomes, Study Design (PICOS) framework (Appendix A), covering three domains: remote care, pregnant women with diabetes, and the IoM's six domains of healthcare quality. Search terms included “remote care”, “remote monitoring”, “telemedicine”, “gestational diabetes”, “pre-gestational diabetes”, “efficiency”, “safety”, “effectiveness”, “timeliness”, “equity”, “patient-centredness”, combined using MeSH terms and Boolean operators. Full search strategies are presented in Appendix A. The search was initially conducted on 25 April 2025 and updated on 30 April 2026.

Studies evaluating remote care interventions for diabetes management during pregnancy, compared with standard in-person or paper-based care, were included. Studies involving non-pregnant populations, prevention of diabetes or GDM, or interventions limited to education, lifestyle, or psychological support were excluded.

No restrictions were placed on diabetes type, diagnostic criteria, or timing of screening, provided participants had a confirmed diagnosis of GDM, type 1 diabetes mellitus (T1DM), or type 2 diabetes mellitus (T2DM). Rare and secondary forms of diabetes were excluded.

Eligible study designs included experimental, controlled, and observational studies. Reviews, conference abstracts, case reports, protocols, and non-full-text articles were excluded. Purely qualitative studies were excluded, although mixed-methods studies reporting relevant outcomes were included. No language restrictions were applied; non-English studies were translated by team members proficient in the relevant language. Full PICOS-based inclusion and exclusion criteria are provided in Appendix A.

This review is registered with the International Prospective Register of Systematic Reviews (PROSPERO; CRD420251024685), follows a previously published protocol,^17^ and adheres to PRISMA 2020 reporting guidelines.^18^

### Data analysis

Search results were screened using Covidence, a web-based systematic review management software.^19^ Title and abstract, and full-text screening were carried out independently by two reviewers (SS, ELE), with any arising conflicts being resolved by consensus. Interrater reliability was assessed using Cohen's Kappa.^20^

Data extraction was independently performed by two reviewers (SS, ELE) using a standardised template. Extracted data included author, publication year, country, study design, population characteristics, sample size, diabetes type, intervention and comparator details, clinical and non-clinical outcomes. Where means and standard deviations were unavailable, established methods were used to estimate these values from medians and interquartile ranges for meta-analysis inclusion.^21^ Clinical measures reported in different units were standardised using recognised conversion factors (e.g., mmol/L to % for HbA1c).

Risk of bias was performed by two independent reviewers (SS, ELE) using the Cochrane Risk of Bias 2 (RoB2) tool for randomised controlled trials (RCTs), and the Risk Of Bias In Non-randomised Studies of Interventions (ROBINS-I) tool for non-randomised studies.^22^^,^^23^ Discrepancies were resolved via consensus.

Outcomes were categorised according to the six IoM domains of healthcare quality: safety, effectiveness, efficiency, timeliness, equity, and patient-centredness. Clinical outcomes included maternal and neonatal outcomes, such as HbA1c and neonatal intensive care unit (NICU) admissions. Efficiency outcomes included healthcare utilisation and costs; timeliness included waiting times; equity included access to care and sociodemographic measures; and patient-centredness included patient experience and usability. A narrative synthesis was conducted by IoM domain.

Meta-analyses were performed on continuous and dichotomous clinical outcomes comparing remote with usual care. Pooled mean differences (MDs) and relative risks (RRs) with corresponding 95% CIs were calculated. Outcomes reported in at least three studies were meta-analysed using random-effects models. Meta-analyses were conducted separately for RCT and non-randomised studies. Sensitivity analyses were performed by risk of bias, remote care type, and diabetes type. Subgroups with a single study were presented but not interpreted as pooled estimates. Publication bias was assessed using funnel plots. Results are presented in forest plots, including effect sizes, 95% CIs, heterogeneity (I^2^), and p-values. All analyses were performed in R version 4.4.1.^24^

### Ethics

This study was a systematic review and meta-analysis of previously published studies and data, therefore, ethical approval and informed consent were not required.

### Role of funding source

The funders had no role in study design, data collection and analysis, decision to publish, or preparation of the manuscript.

## Results

**Fig. 1:**
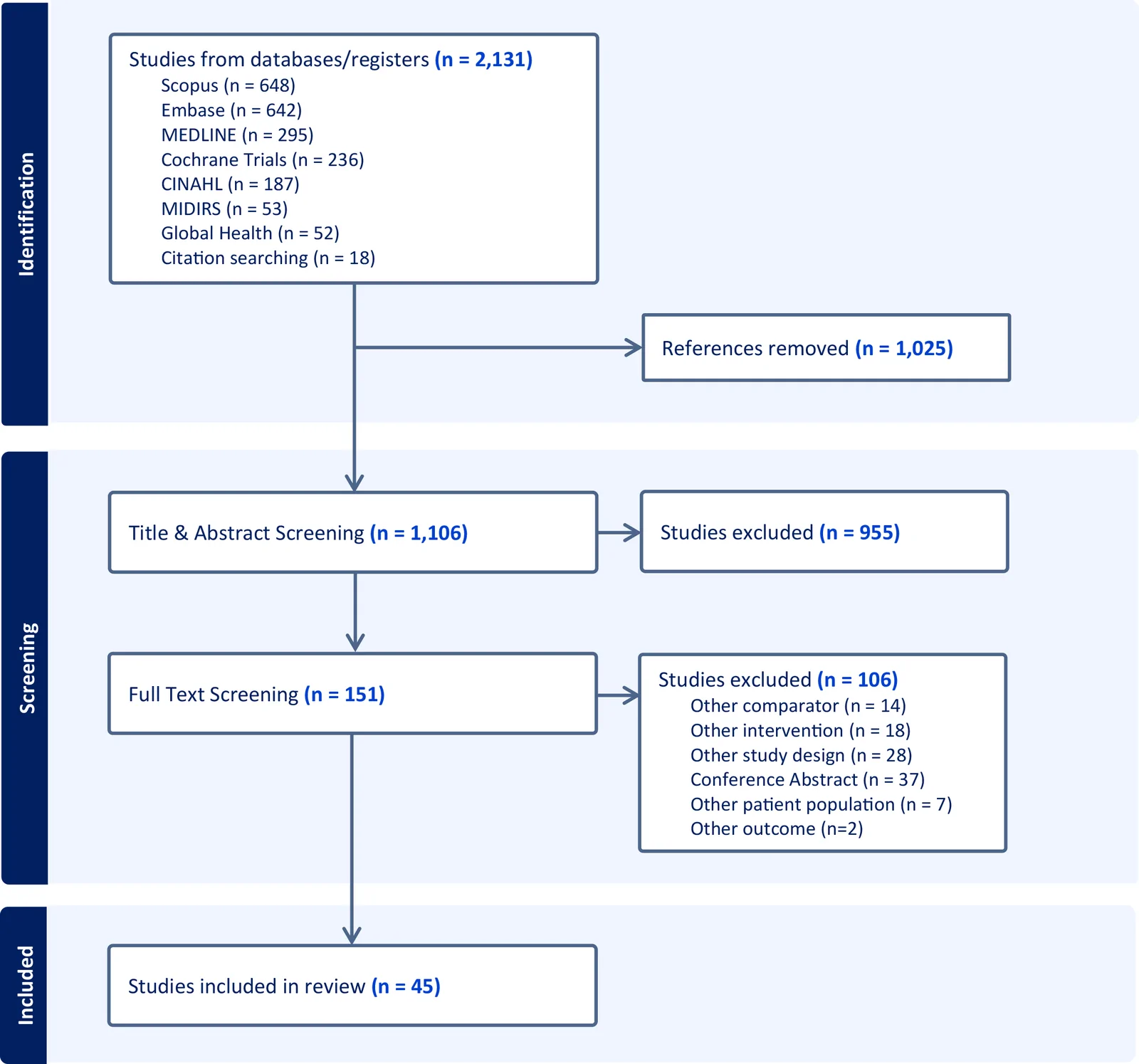
**PRISMA 2020 flow diagram**.^18^

The search yielded 2131 studies, with an additional 18 identified through citation searches. After duplicate removal, 1106 studies underwent screening, of which 45 studies met the inclusion and exclusion criteria and were included in the review. Cohen's Kappa was 0.74 for title and abstract screening and 0.84 for full-text screening, indicating substantial and strong agreement, respectively. The review process is summarised in Fig. 1.

The 45 included studies encompassed 26,562 participants, and were published between 2007 and 2026, with sample sizes ranging from 24 to 9771.^25^, ^26^, ^27^, ^28^, ^29^, ^30^, ^31^, ^32^, ^33^, ^34^, ^35^, ^36^, ^37^, ^38^, ^39^, ^40^, ^41^, ^42^, ^43^, ^44^, ^45^, ^46^, ^47^, ^48^, ^49^, ^50^, ^51^, ^52^, ^53^, ^54^, ^55^, ^56^, ^57^, ^58^, ^59^, ^60^, ^61^, ^62^, ^63^, ^64^, ^65^, ^66^, ^67^, ^68^, ^69^ Study designs comprised randomised controlled trials (RCTs; n = 20, 44.4%), retrospective studies (n = 18, 40%), non-randomised controlled trials (n = 4, 8.9%), prospective cohort studies (n = 2, 4.4%), and observational cohort studies (n = 1, 2.2%). Studies were geographically diverse: most were from China (15.6%), the United States (11.1%), and Canada (8.9%), with additional studies from Europe, the Middle East, Oceania, and Asia. Most studies (n = 37, 82.2%) included women with GDM only, while the remainder included mixed populations with T1DM and/or T2DM.

Of the 20 RCTs, 10 were classified as low risk and 10 as having some concern. Of the 25 non-randomised studies, 17 were rated as moderate and eight as serious risk. Traffic light plots are presented in Appendix B.

Interventions included continuous glucose monitors (CGMs) (n = 2, 4.4%), mobile health (mHealth) applications (n = 29, 64.4%), web-based systems (n = 6, 13.3%), telemedicine (n = 5, 11.1%), and hybrid telemedicine (n = 3, 6.7%). mHealth apps enabled remote blood glucose monitoring through automated or manual upload of glucose readings, with some also incorporating blood pressure and weight measurements, like Tele-Mum,^34^ or provided lifestyle advice, like Pregnant+.^27^ In several studies, mHealth apps were offered in addition to usual care, serving as a digital alternative to self-monitoring blood glucose (SMBG) using paper-based logs.^35^^,^^39^^,^^44^ Twelve studies used historical cohorts, typically pre-implementation or pre-pandemic cohorts, while the remaining studies compared interventions against standard in-person care and SMBG using paper-logs. The study characteristics are presented in Table 1.

**Table 1:** Study and demographic characteristics of included studies.

Author, year	Country	Design	Diabetes type	Diagnosis criteria	Sample size (I/C)	Enrolment/duration	Intervention	Remote care type	Comparator
Al-ofi et al., 2018^25^	Saudi Arabia	Randomised Controlled Trial	GDM	IADPSG	57 (27/30)	From enrolment (24–28 weeks) to 6 weeks post-delivery	Smartphone-glucometer and a glucomail application to monitor blood glucose and weight, with weekly reporting **Name:** Tele-GDM	mHealth	Usual face-to-face clinic
Amylidi-Mohr et al., 2025^26^	Switzerland	Randomised Controlled Trial	GDM	IADPSG	299 (156/143)	From enrolment (24–28 weeks) to delivery Sept 2021–June 2024	Real-time continuous glucose monitoring via rtCGM **Name:** Dexcom G6	CGM	Self-monitoring of blood glucose
Bartholomew et al., 2015^69^	United States of America	Randomised Controlled Trial	GDM T2DM	Carpenter-Coustan	74 (40/34)	From enrolment (<30 weeks) to delivery Feb 2009–Mar 2010	Cell phone/Internet technology (CIT) system: Bluetooth-enabled wireless transfer of glucose readings from OneTouch meter to a mobile phone, uploaded to a secured website for clinician review **Name:** Confidant CIT system	mHealth	Paper based diary logs
Berezowsky et al., 2024^68^	Canada	Retrospective Cohort Study	GDM	50 g GCT followed by 75 g OGTT	775 (187/588)	From enrolment (24 weeks) to delivery Jan 2017–Dec 2020	Combined in-person and virtual (phone or video) antenatal care via the hospital diabetes-in-pregnancy clinic, with visits scheduled every 1–4 weeks depending on glycaemic control	Hybrid Telemedicine	Pre-COVID-19 cohort of usual in-person clinic
Borgen et al., 2019^27^	Norway	Randomised Controlled Trial	GDM	75 g OGTT	238 (115/123)	From enrolment (<33 weeks) to delivery Oct 2015–Apr 2017	Pregnant + smartphone app for feedback on their blood glucose readings (from glucometer), diabetes education, physical activity, and diet promotion. Used in addition to usual care **Name:** Pregnant+	mHealth	Usual care, paper/diary based logs of SMBG biweekly visits
Bromuri et al., 2016^28^	Switzerland	Randomised Controlled Trial	GDM	NR	24 (12/12)	From enrolment (24–32 weeks) to delivery Feb 2013–Jun 2013	Personal health system via a mobile app and web-browsers for remote monitoring to log blood glucose levels and alert of hypo-/hyper-glycaemic episodes	mHealth	Usual care with regular clinic visits, paper based logs of SMBG
Carral et al., 2023^29^	Spain	Prospective Cohort Study	GDM T1DM T2DM	NR	104 (40/64) GDM: 77 PGDM: 27	From enrolment (<30 weeks) to 6–12 weeks post-delivery	Web-based telemedicine system to submit blood glucose levels with hospital visits every 6–8 weeks **Name**: DiabeTIC	Web-based system	Usual care with in-person biweekly visits, SMBG paper based logs 3–6 times per day
Chatzakis et al., 2022^30^	Greece	Non-Randomised Controlled Trial	GDM	IADPSG	63 (31/32)	From enrolment (24–28 weeks) to delivery Jan 2020–Mar 2022	Digital platform (computer, smartphone and tablet) enabling collaborative online monitoring of blood glucose reading submissions between patients and physicians and timely adaptive care **Name:** STORK	mHealth	Historic cohort of usual care of in-person clinic visits
Cichocka et al., 2024^67^	Poland	Retrospective Cohort Study	GDM	WHO	131 (66/65)	From enrolment (24–28 weeks) to delivery. I: 2020–2021 C: 2018–2019	Telemedicine follow-up at diabetes outpatient clinic, Zabrze: first visit in person, subsequent visits every 3–4 weeks via telephone calls, video consultations and online platforms/mobile applications.	Hybrid Telemedicine	Historic cohort of in-person usual care
Clark et al., 2023^31^	Canada	Retrospective Cohort Study	GDM	NR	503 (208/295)	From enrolment (24–28 weeks) to delivery. Apr 2019–Mar 2021	Virtual appointments (phone, email) during COVID-19 pandemic	Telemedicine	Historic cohort of usual care with biweekly clinic visits
Coveney et al., 2026^66^	Ireland	Retrospective Cohort Study	GDM	ADA	583 (356/227)	From enrolment (24–28 weeks) to delivery. I: Jan–June 2021 C: Jan–June 2019	Advanced midwife practitioner led virtual care pathway with integrated Bluetooth-enabled blood glucose monitors syncing to a cloud-based app providing real-time glucose data to clinicians	mHealth	Historic cohort of in-person usual care
Dalfrà et al., 2009^32^	Italy	Non-Randomised Controlled Trial	GDM T1DM	Carpenter-Coustan	235 (105/130) GDM: 203 PGDM: 32	From enrolment (<30 weeks) to delivery	Transmission of glucometer readings via a telephone-based system (Glucobeep) with two-way voice messaging, physicians reviewed results sent SMS alerts for new messages, in addition they had monthly clinic visits	mHealth	Usual care without telemedicine, biweekly visits
Dolatabadi et al., 2025^65^	Canada	Retrospective Cohort Study	GDM	NR	9511 (4236/5275)	From enrolment (24–28 weeks) to delivery. Jan 2017–Dec 2022	Initial virtual (telephone/video) appointment, follow-up appointments mixed virtual (phone, email, video) and in-person	Hybrid telemedicine	Usual in person care
Fincher et al., 2025^64^	United States of America	Retrospective Cohort Study	T1DM T2DM	Pre-existing diagnosis	50 (25/25)	From enrolment to 6 weeks post-partum I: Feb 2017–Jan 2018 C: June 2016–Jan 2017	Cellular-enabled glucose meter transmitting daily glucose readings to a cloud-based portal	mHealth	Usual in person and paper based logs
Gaulier et al., 2017^33^	France	Retrospective Cohort Study	GDM	French CNGOF/SFD	197 (99/98)	From enrolment (24–28 weeks) to delivery I: Mar 2015–May 2016 C: Jul 2012–Jul 2014	Smartphone app with glucose monitoring (every 48 h), automated alerts, teleconsultation via app/email/phone **Name:** SUIDA	mHealth	Usual care with biweekly in-person diabetes reviews
Given et al., 2015^34^	Northern Ireland	Randomised Controlled Trial	GDM	NICE Guidelines	50 (24/26) I: 21 complete	From enrolment (24–28 weeks) to delivery Jan 2012–May 2013	Telemedicine hub involving weekly automatic transmission of SMBG, weight, and blood pressure weekly readings with remote clinician feedback, in addition to usual care **Name:** Tele-Mum	Telemedicine	Usual care of SMBG (7 readings per day), with biweekly clinic visits
Guo et al., 2019^35^	China	Randomised Controlled Trial	GDM	IADPSG	124 (64/60)	From enrolment (24–28 weeks) to delivery Dec 2015–Dec 2017	Mobile app with nurse-led online coaching and data upload of blood glucose and weight readings, in addition to clinic visits **Name:** Dnurse	mHealth	Usual care of SMBG
Homko et al., 2007^36^^,^^42^	United States of America	Randomised Controlled Trial	GDM	Carpenter-Coustan	63 (34/29) (30/23 analysed)	From enrolment (<33 weeks) to delivery Sept 2004–May 2006	Web-based system for inputting of blood glucose values (≥3 times per week), foetal movement counts, insulin doses and any episodes of hypoglycaemia. Patients received personalised feedback from HCPs, and could message between clinicians via the system **Name:** ITSMyHealthrecord	Web-based system	Usual care of SMBG and paper based logs
Homko et al., 2012^37^	United States of America	Randomised Controlled Trial	GDM	Carpenter-Coustan	80 (40/40)	From enrolment (<33 weeks) to delivery Sept 2007–Nov 2009	Internet-based, IVR phone system with automated reminders, and online communication **Name:** ITSMyHealthrecord	Web-based system	Usual care of SMBG and paper based logs
Huang et al., 2025^38^	China	Retrospective Cohort Study	GDM	WHO	100 (50/50)	From enrolment (24–28 weeks) to delivery Apr 2022–Jun 2023	WeChat app-based for self-management of blood glucose levels control, weight and customisation of intervention plans **Name:** WeChat	mHealth	Usual care
Hyams et al., 2024^39^	United Kingdom	Retrospective Cohort Study	GDM	NICE Guidelines	106 (52/54) (47/51 retained)	From enrolment to delivery Sept 2019–Aug 2021	Mobile application for remote blood glucose monitoring, in addition to non-face-to-face interactions (call, text via app) **Name:** GDm-Health	mHealth	Historic cohort pre-GDm-Health implementation of usual care of SMBG
Kantorowska et al., 2023^40^	United States of America	Retrospective Cohort Study	GDM T2DM	NR	533 (360/173)	From enrolment to delivery Oct 2019–Apr 2021	Remote patient monitoring via device integration with Bluetooth glucometers automatically uploading value to patient's Epic MyChart patient portal or via patient manual entry **Name:** Epic MyChart integration	Web-based system	Usual care of SMBG with paper-based blood glucose logs
Kytö et al., 2022^63^	Finland	Randomised Controlled Trial	GDM	75 g OGTT	148 (76/72)	From enrolment (24–28 weeks) to delivery, March 2021–Jan 2023	eMOM GDM smartphone app integrating continuous glucose monitoring, a wrist–worn activity tracker and an electronic 3-day food diary used 1 week/month **Name:** eMOM GDM app	mHealth	Usual in-person care
Laurie et al., 2023^41^	Australia	Prospective Cohort Study	GDM	75 g OGTT	935 (337/598)	From enrolment (24–28 weeks) to delivery June 2019–June 2021	Remote blood glucose monitoring via a Bluetooth glucometer to a mobile application and culturally tailored/translated educational videos **Name:** M♥THer	mHealth	Pre-implementation usual care cohort
Laurie et al., 2023^41^	Australia	Retrospective Cohort Study	GDM	75 g OGTT	935 (337/598)	From enrolment (24–28 weeks) to delivery June 2019–June 2021	Remote blood glucose monitoring via a Bluetooth glucometer to a mobile application and culturally tailored/translated educational videos **Name:** M♥THer	mHealth	Pre-implementation usual care cohort
Lemelin et al., 2020^42^	Canada	Non-Randomised Controlled Trial	GDM	IADPSG/WHO	161 (80/81)	From enrolment (21–30 weeks) to delivery Feb 2016–May 2017	Telehomecare system for remote blood glucose transmission, online platform, algorithm-guided feedback **Name:** THCa	Web-based system	Usual care in clinic
Leyris et al., 2024^43^	France	Retrospective Cohort Study	GDM	French guidelines	1271 (649/622)	From enrolment to delivery Sep 2015–Dec 2020	Mobile application for manual entry or automatic transfer (via Bluetooth) of blood glucose readings **Name:** myDiabby	mHealth	Historic cohort of usual care pre-myDiabby® implementation
Mackillop et al., 2018^44^	United Kingdom	Randomised Controlled Trial	GDM	IADPSG	203 (101/102)	From enrolment (<35 weeks) to delivery Sept 2013–Jun 2015	Mobile application for remote blood glucose monitoring, with outpatient clinic attendance every 4–8 weeks. Automatic alerts generated of non-adherence to recordings. Midwife review at least 3 times a week. **Name:** GDm-health	mHealth	Usual care of SMBG paper based logs, with outpatient clinic visits every 2–4 weeks
Mclennan et al., 2024^45^	United Kingdom	Retrospective Cohort Study	GDM	NICE Guidelines	5251 (2321/2930)	From enrolment to delivery Apr 2018–Mar 2021	Virtual care model of remote monitoring due to COVID-19 pandemic	Telemedicine	Historic cohort of usual in-person care pre-covid
Miremberg et al., 2018^46^	Israel	Randomised Controlled Trial	GDM	50 g GCT + 100 g OGTT	120 (60/60)	From enrolment (<34 weeks) to delivery May 2016–May 2017	Smartphone application for the submission of daily blood glucose measurement with daily feedback system, in addition to usual care **Name:** GlucoseBuddy	mHealth	Usual care of biweekly in-person clinic visits
Montori et al., 2023^47^	Italy	Observational Cohort Study	GDM	75 g OGTT	60 (27/33)	From enrolment to delivery Feb 2018–Aug 2019	Automatic transfer of SMBG levels to a virtual cloud via a smartphone application, and monthly telemedicine visits per month	mHealth	Usual care with outpatient clinics every 2 or 3 weeks
Munda et al., 2023^48^	Slovenia	Randomised Controlled Trial	GDM	IADPSG	105 (53/52)	From enrolment (<30 weeks) to delivery Mar 2020–Oct 2021	Sending of SMBG readings via a smartphone application, follow-up done via telemedicine	mHealth	Usual care of in-person monthly outpatient visits
Peleg et al., 2017^49^	Spain	Retrospective Cohort Study	GDM	75 g OGTT	267 (20/247)	From enrolment to delivery. I: Apr–Dec 2015 C: 2010–2013	Personalised decision-support system (DSS) used by patients and their care providers with Bluetooth-connected glucose meters **Name:** MobiGuide	mHealth	Historic cohort of usual care of face-to-face outpatient visits
Pérez-Ferre et al., 2010^50^	Spain	Randomised Controlled Trial	GDM	Carpenter-Coustan	97 (49/48)	From enrolment (<28 weeks) to delivery Jun–Dec 2008	Mobile phone application for the transmission of SMBG values to the central database via SMS	mHealth	Usual care of SMBG, monthly face-to-face outpatient clinic visits
Poncelet et al., 2024^51^	France	Retrospective Cohort Study	GDM	French Guidelines	644 (349/295)	From enrolment to delivery I: Jan–Dec 2021 C: Jan 2013–Jun 2015	Telemonitoring of blood glucose readings via a smartphone app **Name:** myDiabby	mHealth	Usual care of SMBG using a diary, biweekly visits
Rasekaba et al., 2018^52^	Australia	Randomised Controlled Trial	GDM	75 g OGTT	95 (61/34)	From enrolment (<36 weeks) to delivery 2014–2016	Telemedicine using a Web-based portal, Online Health Portfolio (OHP) for data sharing of SMBG readings and communication between patients and clinicians **Name:** TeleGDM/OHP	Web-based system	Usual face-to-face care
Shashikumar et al., 2022^53^	New Zealand	Retrospective Cohort Study	GDM T1DM T2DM	New Zealand guidelines	366 (179/187)	From enrolment to delivery Mar 2019–May 2020	Teleclinic phone based consultations due to COVID-19 pandemic	Telemedicine	Historic cohort of usual in-person care
Sung et al., 2021^54^	United States of America	Retrospective Cohort Study	PGDM	N/A	593 (172/421)	Full pregnancy 2013–2016	Statewide telemedicine programme	Telemedicine	Usual in-person care
Syrop et al., 2020^55^	United States of America	Retrospective Cohort Study	GDM, T1DM T2DM	ADA or OGTT	251 (129/122)	From enrolment to delivery Apr 2014–Jun 2018	Integrated practice unit with remote glucose monitoring (cellular-enabled glucometers) **Name:** Telcare™	mHealth	Historic cohort of usual care
Tian et al., 2021^56^	China	Randomised Controlled Trial	GDM	IADPSG	309 (133/136)	From enrolment (23–30 weeks) to delivery Jun 2019–Dec2019	WeChat-based group for glucose management via SMBG readings with weekly education and peer support, in addition to usual care **Name:** WeChat	mHealth	Usual care of SMBG with biweekly prenatal care visits
Wang et al., 2025^57^	China	Non-randomised Controlled Trial	GDM	IADPSG	56 (26/26)	From enrolment (24–28 weeks) to delivery	Personalised WeChat mini-program for glucose management, where patients would upload blood glucose levels, exercise levels, and diets **Name:** WeChat	mHealth	Usual care
Wei et al., 2016^58^	China	Randomised Controlled Trial	GDM	ADA/IADPSG	106 (51/55)	From enrolment (24–28 weeks) to delivery Sept 2011–Dec 2012	Continuous glucose monitoring system recording values 4 times a day **Name:** Gold Medtronic MiniMed	CGM	Usual care of SMBG with 4 daily readings
Wen et al., 2025^60^	China	Randomised Controlled Trial	GDM	75 g OGTT	225 (116/109)	From enrolment to delivery	Bespoke medical Internet-of-Things (mIoT) platform: smartphone application allowing real-time logging of glucose, diet, exercise, and weight	mHealth	Usual care of paper-based logs
Yew et al., 2021^61^	Singapore	Randomised Controlled Trial	GDM	WHO/75 g OGTT	340 (170/170)	From recruitment (12–30 weeks) to delivery Sept 2017–Nov 2018	SMBG via Aina/Aina Mini glucometer feeding directly into the app; integrated diet/SMBG/physical-activity/weight tracking, automated coaching and behaviour-change cues **Name**: Habits-GDM app	mHealth	Usual in-person and SMBG paper logs with 2–4 weekly follow-ups
Zhuo et al., 2022^59^	China	Randomised Controlled Trial	GDM	ADA/IADPSG	124 (58/61)	From enrolment (24–35 weeks) to delivery Jan 2018–Oct 2020	Pharmacist-led diabetes self-management smartphone of uploaded SMBG values, insulin doses, diet, physical exercise, and two-way real-time consultation, hyper-/hypo-glycaemia alerts, in addition to usual biweekly outpatient clinic visits Name: CMC	mHealth	Usual care of SMBG on a diary 3 times a week with biweekly outpatient visits

Abbreviations: ADA = American Diabetes Association; C = Control; CMC = Continuing Medical Care; CNGOF = Collège National des Gynécologues et Obstétriciens Français; CGM = Continuous Glucose Monitoring; DSS = Decision Support System; GCT = Glucose Challenge Test; GDM = Gestational Diabetes Mellitus; GW = Gestational Weeks; HCP = Healthcare Professional; I = Intervention; IADPSG = International Association of the Diabetes and Pregnancy Study Groups; IVR = Interactive Voice Response; mHealth = Mobile Health; N/A = Not Applicable; NICE = National Institute for Health and Care Excellence; NR = Not Reported; OGTT = Oral Glucose Tolerance Test; OHP = Online Health Portfolio; PGDM = Pregestational Diabetes Mellitus; PSM = Propensity Score Matched; SMBG = Self-Monitoring of Blood Glucose; SFD = Société Francophone du Diabète; T1DM = Type 1 Diabetes Mellitus; T2DM = Type 2 Diabetes Mellitus.

Effectiveness was the most frequently assessed IoM domain (n = 44, 97.8%), while timeliness was the least evaluated (n = 3, 6.7%). Safety, efficiency, patient-centredness, and equity were assessed in 34 (75.6%), 22 (48.9%), 14 (31.1%), and 11 (24.4%) studies, respectively. Due to the heterogeneity of the studies and the complexity of diabetes management during pregnancy, a wide range of clinical outcomes were reported. Over 30 distinct maternal and neonatal clinical outcomes were reported across studies.

**Fig. 2:**
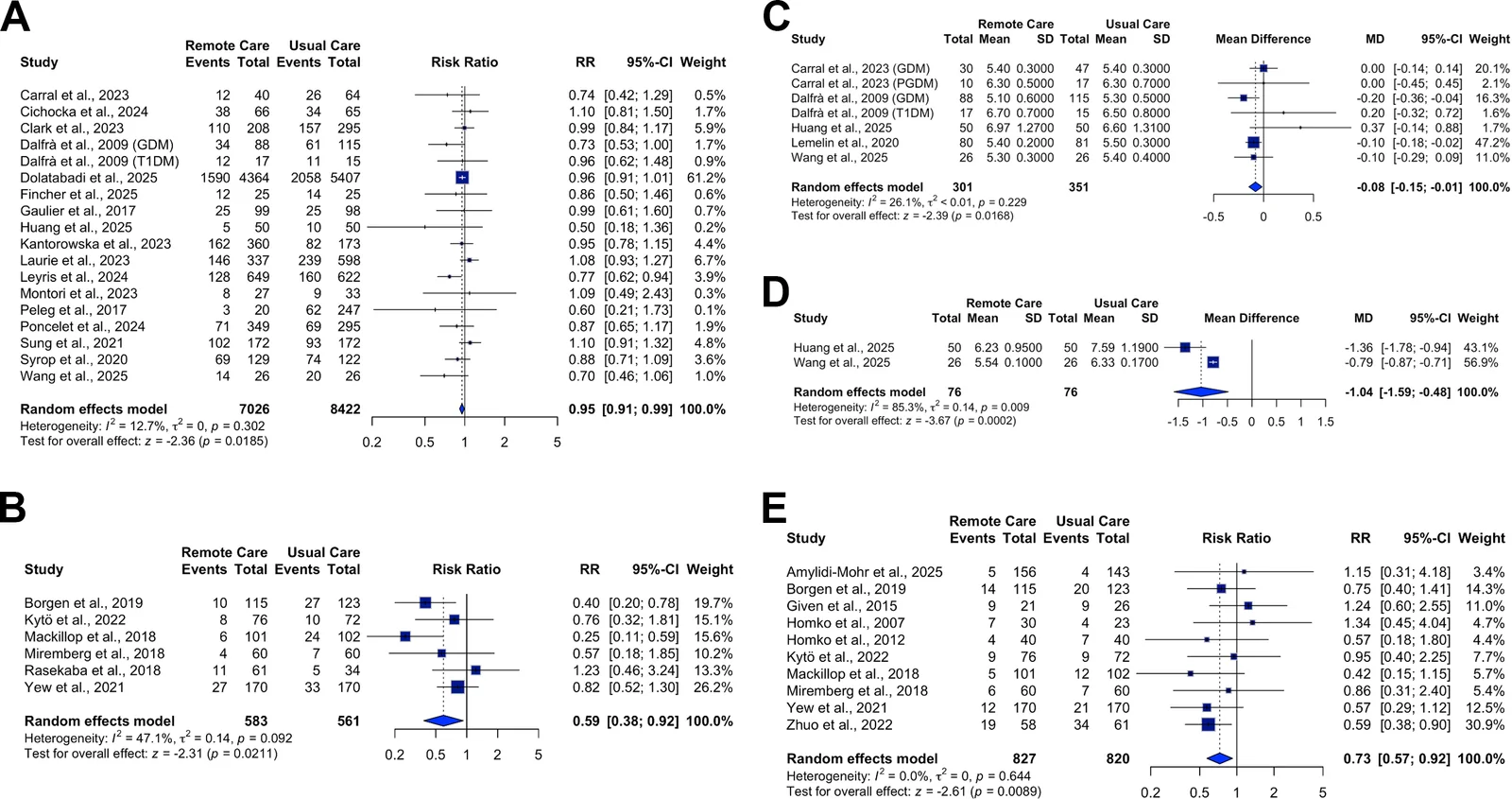
**Forest plots comparing remote vs usual care**. **(A)** Unspecified caesarean section in non-randomised studies; **(B)** emergency caesarean section in RCTs; **(C)** postpartum HbA1c (%) in non-randomised studies; **(D)** postpartum 2-h post-prandial glucose (mmol/L) in non-randomised studies; and **(E)** neonatal intensive care unit (NICU) admission in RCTs. Abbreviations: RCT = Randomised Controlled Trial; NICU = Neonatal Intensive Care Unit.

In non-randomised studies, remote care was associated with lower rates of unspecified caesarean sections (RR 0.95, 95% CI 0.91–0.99, *k =* 18), reduced postpartum 2-h post-prandial glucose (PPG) levels (MD −1.04 mmol/L, 95% CI −1.59 to −0.48; *k =* 2) and lower HbA1c (MD −0.08%, 95% CI −0.15 to −0.01, *k =* 7) (Fig. 2). No significant differences were observed for gestational weight gain (GWG), elective caesarean sections, vaginal birth, pharmacological initiation, hypertensive disorders, or fasting plasma glucose (Table 2).

**Table 2:** Pooled effects of maternal and neonatal effectiveness outcomes of remote vs usual care.

	Outcome	Study design	*k*	Observations	Pooled effect			Heterogeneity I^2^
					Effect size	95% CI	p-value	
Maternal Outcomes	Elective c-section	RCT	6	1144	RR = 1.00	0.75, 1.34	0.9901	16.7%
		Non-RCT	5	6616	RR = 1.02	0.83, 1.26	0.8471	55.0%
	C-section (not specified)	RCT	10	1299	RR = 1.01	0.83, 1.22	0.9350	50.8%
		Non-RCT	18	15,448	RR = 0.95	0.91, 0.99	0.0185	12.7%
	Vaginal spontaneous	RCT	8	1690	RR = 1.06	0.97, 1.15	0.1933	24.4%
		Non-RCT	12	18,895	RR = 1.01	0.96, 1.07	0.6520	48.8%
	Vaginal operative	RCT	6	1324	RR = 1.05	0.64, 1.72	0.7069	36.3%
		Non-RCT	8	8112	RR = 0.96	0.72, 1.29	0.7799	44.3%
	Gestational weight gain (kg)	RCT	11	1612	MD = −0.67	−1.35, 0.02	0.0559	91.4%
		Non-RCT	7	854	MD = −0.99	−2.01, 0.02	0.0553	27.7%
	Insulin started	RCT	10	1462	RR = 1.07	0.74, 1.54	0.7168	59.8%
		Non-RCT	18	20,947	RR = 1.04	0.94, 1.16	0.4204	71.1%
	Metformin started	RCT	2	561	RR = 0.66	0.32, 1.37	0.2648	0.0%
		Non-RCT	6	17,171	RR = 1.04	0.82, 1.25	0.7638	93.0%
	Pre-eclampsia/Eclampsia	RCT	2	419	RR = 1.34	0.52, 3.46	0.5415	0.0%
		Non-RCT	6	2918	RR = 0.97	0.38, 2.00	0.7477	62.6%
	GHTN	RCT	3	264	RR = 0.93	0.15, 5.60	0.9357	0.0%
		Non-RCT	8	2180	RR = 1.02	0.67, 1.55	0.9375	0.0%
	Preeclampsia/Eclampsia/GHTN	RCT	9	1387	RR = 1.10	0.68, 1.77	0.7099	0.0%
		Non-RCT	11	8702	RR = 0.94	0.71, 1.26	0.6894	9.0%
	Post-partum HbA1c (%)	RCT	9	1151	MD = −0.12	−0.29, 0.04	0.1430	82.9%
		Non-RCT	7	652	MD = −0.08	−0.15, −0.01	0.0168	26.1%
	Post-partum FPG (mmol/L)	RCT	8	766	MD = −0.53	−1.44, 0.37	0.2467	99.4%
		Non-RCT	3	349	MD = −0.34	−0.86, 0.18	0.2046	95.0%
	Post-partum 2-hr PPG (mmol/L)	RCT	6	522	MD = −0.27	−0.60, 0.07	0.1182	64.1%
		Non-RCT	2	152	MD = −1.04	−1.59, −0.48	0.0002	85.3%
Neonatal Outcomes	Birthweight (grams)	RCT	11	1173	MD = 1.27	−75.59, 78.12	0.9742	57.7%
		Non-RCT	17	11,497	MD = −16.52	−36.43, 3.38	0.1038	10.8%
	Macrosomia	RCT	10	1785	RR = 0.77	0.55, 1.06	0.1121	0.0%
		Non-RCT	12	13,088	RR = 0.96	0.75, 1.22	0.7150	0.0%
	SGA	RCT	2	308	RR = 1.91	0.45, 8.03	0.3769	0.0%
		Non-RCT	5	15,538	RR = 1.02	0.92, 1.12	0.7446	0.0%
	LGA	RCT	9	1211	RR = 1.02	0.75, 1.39	0.9004	22.0%
		Non-RCT	13	20,019	RR = 0.94	0.84, 1.06	0.3352	38.7%
	GA at delivery	RCT	11	1329	MD = −0.02	−0.21, 0.17	0.8167	55.6%
		Non-RCT	16	10,544	MD = −0.03	−0.12, 0.05	0.4642	37.4%
	PTB	RCT	11	1866	RR = 0.97	0.67, 1.42	0.8922	2.2%
		Non-RCT	11	5063	RR = 0.88	0.71, 1.09	0.2287	0.5%

Abbreviations: C-section = Caesarean Section; FPG = Fasting Plasma Glucose; PPG = Post-Prandial Glucose; SGA = Small for Gestational Age; LGA = Large for Gestational Age; GA = Gestational Age; GHTN = Gestational Hypertension; PTB = Pre-term Birth; RR = Relative Risk; MD = Mean Difference; CI = Confidence Interval.

GDM-restricted analyses yielded similar results for postpartum HbA1c (MD −0.09, 95% CI −0.15 to −0.02; k = 5) and postpartum 2-h PPG levels (MD −1.04, 95% CI −1.59 to −0.48; k = 2) (Table D1).

Sensitivity analyses by intervention type identified several associations in non-randomised studies: telemedicine was associated with increased elective caesarean section risk (RR 1.18, 95% CI 1.06–1.30; k = 2) and reduced operative vaginal birth risk (RR 0.76, 95% CI 0.65–0.90; k = 2); hybrid telemedicine with increased spontaneous vaginal birth (RR 1.05, 95% CI 1.01–1.09; k = 3); mHealth with lower GWG (MD −1.33 kg, 95% CI −2.61 to −0.06; k = 5) and unspecified caesarean section (RR 0.87, 95% CI 0.77–0.98; k = 12); and web-based systems with reduced hypertensive disorders (RR 0.66, 95% CI 0.44–1.00; k = 3).

Low-risk study restriction yielded broadly consistent findings, with remote care remaining associated with lower GWG in non-randomised studies (MD −1.27 kg, 95% CI −2.53 to −0.01; k = 5) and elective caesarean sections (RR 1.15, 95% CI 1.05–1.27; k = 4). In RCTs, postpartum 2-h PPG remained lower with remote care (MD −0.29, 95% CI −0.43 to −0.16; k = 2), with no other significant differences observed (Table F1).

No significant differences were observed for birthweight, macrosomia, small or large for gestational age, gestational age at delivery, or preterm birth (Table 3), including GDM-restricted analyses (Appendix D; Table D2). Sensitivity analyses by intervention type showed reduced LGA risk with hybrid telemedicine in non-randomised studies (RR 0.82, 95% CI 0.72–0.93; k = 2) and reduced macrosomia with mHealth in RCTs (RR 0.67, 95% CI 0.46–0.98; k = 6). No differences were observed when excluding high-risk studies (Tables E2 and F2).

**Table 3:** Pooled effects of maternal and neonatal safety outcomes of remote vs usual care.

	Outcome	Study design	*k*	Observations	Pooled effect			Heterogeneity I^2^
					Effect size	95% CI	p-value	
Maternal Outcomes	Emergency c-section	RCT	6	1144	RR = 0.59	0.38, 0.92	0.0211	47.1%
		Non-RCT	4	6616	RR = 0.99	0.85, 1.16	0.8960	16.4%
	Induction of Labour	RCT	4	805	RR = 0.90	0.76, 1.06	0.1960	14.5%
		Non-RCT	8	9729	RR = 1.05	0.90, 1.21	0.5418	69.7%
	PROM	RCT	4	551	RR = 1.34	0.44, 4.08	0.6081	10.4%
		Non-RCT	1	161	RR = 0.76	0.34, 1.70	0.5039	N/A
	Polyhydramnios	RCT	3	538	RR = 1.08	0.74, 1.57	0.7006	21.6%
		Non-RCT	1	63	RR = 1.03	0.07, 15.79	0.9818	N/A
Neonatal Outcomes	NICU	RCT	10	1647	RR = 0.73	0.57, 0.92	0.0089	0.0%
		Non-RCT	10	18,558	RR = 0.94	0.84, 1.05	0.2507	35.4%
	Jaundice	RCT	8	1110	RR = 0.97	0.72, 1.33	0.6539	0.0%
		Non-RCT	8	3625	RR = 1.00	0.57, 1.76	0.9958	68.7%
	Hypoglycaemia	RCT	12	1736	RR = 0.95	0.74, 1.23	0.6951	0.0%
		Non-RCT	11	9125	RR = 0.74	0.40, 1.37	0.3301	84.5%
	Respiratory Distress	RCT	7	1159	RR = 0.84	0.60, 1.17	0.2965	6.5%
		Non-RCT	7	6993	RR = 1.20	0.87, 1.66	0.2668	5.8%
	Shoulder Dystocia	RCT	4	1038	RR = 0.58	0.18, 1.89	0.3706	22.1%
		Non-RCT	9	9508	RR = 0.81	0.36, 1.81	0.6128	56.4%
	Stillbirth	RCT	2	419	Not defined			
		Non-RCT	5	16,376	RR = 0.73	0.40, 1.33	0.3027	0.0%

Abbreviations: C-section = Caesarean Section; PROM = Premature Rupture of Membranes; NICU = Neonatal Intensive Care Unit; RR = Relative Risk; CI = Confidence Interval.

In RCTs, remote care was associated with a lower risk of emergency caesarean section (RR 0.59, 95% CI 0.38–0.92; k = 6), driven primarily by mHealth interventions (RR 0.53, 95% CI 0.34–0.85; k = 5); this was not observed in non-randomised studies. No significant effects were observed for induction of labour, PROM, or polyhydramnios, and no differences emerged in GDM-restricted or low-risk sensitivity analyses (Tables D3, E3, F3).

Remote care was associated with reduced NICU admission risk in RCTs (RR 0.73, 95% CI 0.57–0.92; k = 10), consistent across mHealth subgroups (RR 0.64, 95% CI 0.49–0.85; k = 6). No significant effects were observed for jaundice, hypoglycaemia, shoulder dystocia, stillbirth, or respiratory distress, except in GDM-restricted non-RCTs where remote care was associated with increased respiratory distress risk (RR 1.34, 95% CI 1.00–1.78; k = 5) (Tables D4, E4, F4).

Funnel plots, pooled effects, sensitivity analyses, and forest plots for effectiveness and safety-related outcomes are presented in Appendix D–H.

Four of six cost-reporting studies found remote care to be cost-effective^42^^,^^44^^,^^54^^,^^62^; one reported higher costs due to an additional programme cost of US$1942 per delivery,^55^ and one found similar appointment costs but higher unplanned care costs in the intervention group.^52^ Of 20 studies reporting appointment frequency or contact time, nine reported greater interaction frequency with remote care and six reported fewer visits or reduced contact time^29^^,^^30^^,^^35^^,^^42^^,^^44^^,^^54^; two showed improved attendance^31^^,^^34^ and two showed reduced consultation time.^47^^,^^50^ A detailed summary is presented in Appendix C.

No difference was observed in time to medication initiation (insulin or metformin) between GDm-health and usual care (I: 20 [13–33] vs C: 19 [8.5–34.5], p = 0.59),^39^ or between web-based systems and usual care (I: 28.0 (SD 17.8) vs C: 29.0 (SD 10.4)).^42^ However, women in the virtual care clinic experienced faster treatment initiation from diagnosis (within 5 days: 42.6% vs 58.8%, p < 0.001, within 10 days: 77.3% vs 94.9%, p < 0.001).^66^

Of 14 studies evaluating patient-centredness outcomes, two reported neutral effects and one positive effects on quality of life. Dalfrà et al. found no significant post-delivery differences in SF-36 scores.^32^ Homko et al. (2007) reported improved self-efficacy (p = 0.04), but their 2012 study found no differences in anxiety.^36^^,^^37^ All 11 studies evaluating experience, usability, and acceptability reported positive findings or outcomes comparable to usual care. Users of GDm-health, MobiGuide, and Pregnant + reported high satisfaction, willingness to reuse, and greater engagement (p < 0.01).^27^^,^^28^^,^^47^ During COVID-19, 93% rated virtual care as “good” or “excellent”, with 90% noting cost/time savings and minimal technical issues.^31^ Shashikumar et al. reported 89% high satisfaction, though some noted communication delays.^53^ CGM users preferred rtCGM over SMBG for ease of use (p = 0.001).^26^ Bartholomew et al. found strong patient preference for remote care (68.9% vs 24.3%, p < 0.001), with higher scores across ease of use, motivation, and personalisation.^69^ Cichocka et al. reported that over 60% of women preferred teleconsultation without perceiving pandemic-related care quality losses.^67^

Amylidi-Mohr et al. reported a multi-ethnic cohort (one-third non-white) with no significant differences in outcomes by ethnicity,^26^ no other studies quantified ethnicity-based impact. Several mHealth interventions incorporated cultural and linguistic adaptations, including multilingual support in Pregnant+ (Norwegian, Urdu, Somali), STORK (Greek, English), and M♥THER (culturally tailored videos).^27^^,^^30^^,^^41^ Dolatabadi et al. found that women attending virtual initial appointments were more likely to reside in rural areas and have higher material deprivation scores, suggesting remote care may reach those facing significant attendance barriers.^65^

Digital access barriers were also documented. Carral et al. noted that some women lacked home Internet access, though all affected participants were in the control group.^29^ In Homko et al. (2012), 18% of the intervention group lacked home Internet access but used an interactive voice response system instead.^37^ Tele-Mum faced slow or unreliable data transmissions in rural areas due to poor mobile reception,^34^ while SMART-GDM excluded women without smartphone access or unable to navigate an app.^61^ Conversely, Dalfrà et al.‘s telephone-based system required no technological expertise or computer literacy.^32^ The STORK platform reduced clinic visits (4.1 to 2.9; p < 0.05), improving access for patients facing travel or logistical barriers.^30^ Laurie et al. estimated per-patient savings of AU$566.56 under the digital model, driven by reductions in lost wages, childcare, and travel costs.^62^

## Discussion

This systematic review synthesised evidence on remote care for pregnant women with diabetes, a population facing well-established maternal and neonatal risks and growing global prevalence; effective management can improve outcomes and reduce long-term disease burden and healthcare costs.^70^, ^71^, ^72^ Remote care delivered outcomes largely comparable to usual care across most maternal and neonatal measures. In non-randomised studies, remote care was associated with lower unspecified caesarean section rates (RR 0.95, 95% CI 0.91–0.99), lower postpartum HbA1c (MD −0.08%, 95% CI −0.15 to −0.01), and lower postpartum 2-h PPG (MD −1.04 mmol/L, 95% CI −1.59 to −0.48). In RCTs, remote care was associated with reduced emergency caesarean section risk (RR 0.59, 95% CI 0.38–0.92) and NICU admission (RR 0.73, 95% CI 0.57–0.92). No significant differences were observed for GWG in either non-randomised or RCTs, nor in RCTs for unspecified caesarean sections or postpartum HbA1c. Patient satisfaction was consistently high. Evidence on insulin and metformin initiation was mixed. Efficiency outcomes, such as appointment frequency and costs, were mixed. Equity measures were rarely formally reported, though several interventions incorporated cultural and linguistic adaptations. Key findings are summarised in Panel 1.

Panel 1 **Summary of the review's key findings across the six Institute of Medicine domains of healthcare quality**

**Effectiveness**

• Remote care was associated with lower post-partum HbA1c (mean difference [MD] −0.08%; p = 0.0168; k = 7), lower post-partum 2-h post-prandial glucose levels (MD −1.04 mmol/L; p = 0.0002; k = 2), and lower unspecified caesarean section rates (relative risk [RR] 0.95; p = 0.0185; k = 18) in non-randomised studies but no significant differences were observed in randomised controlled trials (RCTs).
• Sensitivity analyses by remote care type showed, in non-randomised studies, reduced risk of operative vaginal birth with telemedicine (RR 0.76; p = 0.0010; k = 2), lower unspecified caesarean section (RR 0.87; p = 0.0209; k = 12) and lower gestational weight gain (MD −1.33 kg; p = 0.0407; k = 5) with mHealth, and increased spontaneous vaginal birth with hybrid telemedicine (RR 1.05; p = 0.0131; k = 3).
• No significant effects were observed for spontaneous vaginal birth, elective caesarean section, gestational weight gain, insulin or metformin use, gestational hypertension, pre-eclampsia, or fasting plasma glucose.
• No significant effects were observed for gestational age at delivery, preterm birth, birthweight, macrosomia, large for gestational age, or small for gestational age.

**Safety**

• Remote care was associated with a reduced risk of emergency caesarean section in RCTs (RR 0.59; p = 0.0211; k = 6), driven primarily by mHealth interventions (RR 0.53; p = 0.0077; k = 5); no significant difference was observed in non-randomised studies.
• Remote care was associated with lower NICU admission rates in RCTs (RR 0.73; p = 0.0089; k = 10), consistent in the mHealth subgroup; the effect was not significant in non-randomised studies.
• No significant effects were observed for induction of labour, premature rupture of membranes, or polyhydramnios.
• No significant effects were observed for neonatal hypoglycaemia, jaundice, shoulder dystocia, respiratory distress, or stillbirth.

**Efficiency**

• Mixed results seen with respect to cost, four of six studies demonstrated cost savings with remote care, one found similar costs and one reported higher overall costs
• Mixed results with respect to appointment frequency; of 20 studies reporting appointment frequency or contact time, nine reported a greater number of interactions with remote care while six reported fewer visits or reduced contact time. Two studies reported improved attendance and two reported reduced consultation time.

**Patient-Centeredness**

• Of 14 studies assessing patient-centredness, three reported quality of life outcomes; two showed a neutral effect and one demonstrated improved self-efficacy
• All 11 studies reporting experience, usability, or acceptability outcomes found high or comparable satisfaction, ease of use, and willingness to reuse remote systems, citing convenience, flexibility, and perceived safety. One study reported that patients preferred continuing virtual care (telemedicine) post-pandemic.

**Timeliness**

• No significant difference in time to medication initiation (insulin, metformin) was observed in two studies
• One study reported faster treatment initiation from diagnosis in the virtual care group.

**Equity**

• Of 11 studies assessing equity, three remote care interventions incorporated language and cultural adaptations to improve accessibility, however, there was no formal assessment of their effect on outcomes
• Rural patients faced connectivity barriers, but telephone-based systems mitigated access gaps
• One study found that women attending virtual initial appointments were more likely to reside in rural areas and have higher material deprivation scores, suggesting virtual care may reach underserved groups.
• Overall, formal evaluation of the impact of digital, language, and cultural barriers on outcomes remained limited.

Remote care was not associated with a higher risk of elective caesarean, however, substantial variation was observed at the study level, likely reflecting national guidelines and clinical practice differences. In GDM, elective caesareans are often precautionary due to macrosomia and shoulder dystocia risks.^73^ However, over 75% of studies did not specify caesarean type, i.e. elective vs emergency, limiting subgroup analysis and generalisability.

Remote care was not associated with a significant reduction in GWG in either non-randomised studies or RCTs. This contrasts with prior evidence that remotely delivered, evidence-based programmes can prevent excessive GWG and improve insulin resistance markers, though findings across reviews remain mixed.^74^ Reductions did emerge in the mHealth subgroup (MD −1.33 kg, 95% CI −2.62 to −0.06) and when analysis was restricted to low-risk studies (MD −1.27 kg, 95% CI −2.53 to −0.01), however, both are based on small numbers of studies and should not be regarded as confirmatory. The absence of an overall effect may partly reflect the failure to account for individualised BMI-based targets, alongside heterogeneity in how weight was measured and in the behavioural content of interventions. Other studies have demonstrated that mHealth and telemedicine tools can reduce GWG and postpartum weight/BMI in GDM populations,^75^^,^^76^ highlighting the importance of structured behavioural management incorporating goal-setting, self-monitoring feedback loops, dietary counselling, physical activity guidance, and motivational support, aligned with behaviour change frameworks such as the COM-B model.

Improvements in postpartum HbA1c and 2-h PPG in non-randomised studies are plausible: continuous glucose monitoring, app-based logging, and clinician feedback loops may reinforce self-monitoring habits and health literacy that persist beyond delivery. Structured nutritional counselling and automated alerts embedded in several interventions may further contribute. However, these differences are of modest absolute magnitude and were not replicated in RCTs, where neither postpartum HbA1c (MD −0.05%, p = 0.14) nor 2-h PPG (MD −0.26 mmol/L, p = 0.12) reached significance. Selection bias in non-randomised designs, where more digitally engaged and adherent women may self-select into remote care, likely contributes to the more favourable findings observed. Furthermore, these differences in values may reflect the natural history of glucose dysregulation following pregnancy. These associations should therefore be interpreted with caution, though any postpartum glycaemic advantage is clinically meaningful given the substantially elevated lifetime risk of T2DM in this population.

A 2017 Cochrane Review of RCTs in GDM reported no significant differences in maternal or neonatal outcomes (pre-eclampsia, caesarean, induction, hypoglycaemia, LGA) with telemedicine.^77^ The present findings are consistent with this, showing no evidence of inferiority for remote care and extending the evidence base to broader intervention types and outcome domains. Notably, reduced emergency caesarean section and NICU admission rates in RCTs represent clinically important safety signals, given the complications and distress associated with both outcomes.^78^ However, NICU admission definitions vary considerably across institutions, and future studies should adopt standardised criteria to permit valid comparisons.

Heterogeneity in MDT composition represents an underappreciated source of clinical variability. Structured dietetic input is associated with improved glycaemic outcomes and reduced pharmacological need in GDM, yet MDT composition was inconsistently reported across included studies, limiting direct comparability.^79^ Technology-based interventions also appear more effective when designed around end-user needs and embedded within behavioural support frameworks, rather than deployed as standalone tools.^80^

Though cost data were limited, four of six studies reported savings, consistent with previous findings in related populations. Remote models may reduce costs by minimising non-attendance, travel time, and treatment delays, though setup costs may offset short-term gains.^81^ For example, a dietitian-led phone programme for GDM prevention demonstrated incremental cost-effectiveness of AUD$105.^82^ Homko et al. noted improved efficiency as a key benefit of telemedicine.^36^ While some studies report setup costs,^55^ these may be offset by long-term savings.

Timeliness evidence was sparse; two studies reported no difference in time to medication initiation, while one found faster treatment initiation in the virtual care group, suggesting potential for earlier intervention as service demand grows. Standard GDM management relies on SMBG and paper logs reviewed at clinic visits. As diabetes in pregnancy increases, so does demand for specialised care,^83^, ^84^, ^85^ placing significant strain on healthcare resources.^86^ Remote care, especially asynchronous models, may ease this burden by enabling earlier interventions, potentially reducing adverse outcomes.^33^^,^^34^^,^^49^

Patient satisfaction was consistently high across study designs, intervention types, and settings, supporting acceptability and willingness to engage with remote systems. Accessibility and equity remain important considerations, for example, lack of logbooks in patients’ first languages is a barrier to GDM management,^87^ and interventions without multilingual functionalities may similarly restrict patient engagement and utility. Several interventions offered multilingual support and culturally adapted content,^27^^,^^30^ and one study found that virtual attendees were more likely to reside in rural areas with higher material deprivation scores, suggesting remote care may reach underserved groups. However, digital literacy gaps, limited internet access, and poor network coverage in rural areas remain barriers to equitable implementation that require proactive design solutions.

The available evidence is predominantly derived from GDM populations selectively offered remote care, often those with lower clinical complexity. This limits generalisability to higher-complexity populations such as those with T1DM or insulin-dependent T2DM in pregnancy. These findings should not be interpreted as supporting remote care as uniformly safe for all pregnant women with diabetes without prior risk stratification. The substantial clinical and methodological heterogeneity across included studies means that pooled estimates should be interpreted with appropriate caution; findings suggest no clear inferiority of remote care rather than equivalence per se. Amylidi-Mohr et al. found that SMBG was more effective than real-time CGM for certain glycaemic outcomes in GDM, underscoring that the evidence base for CGM in GDM is still evolving and that consensus on glycaemic targets and management protocols for CGM in GDM and T2DM in pregnancy has not yet been established.

This review has several strengths, it followed a predefined, peer-reviewed protocol,^88^ and to the authors’ knowledge, is the first to evaluate the remote care in pregnant women with diabetes across all six IoM healthcare quality domains. Previous reviews have evaluated telemedicine in this population but did not cover all six domains.^89^, ^90^, ^91^, ^92^ Using this framework allowed for a structured assessment across key dimensions, as seen in prior virtual care evaluations.^12^^,^^93^, ^94^, ^95^ Subgroup analyses by intervention type, diabetes type, and study design reduced confounding, and the large sample size (n = 26,562) strengthened statistical power.

There are several limitations to consider. Significant clinical and methodological heterogeneity was present across studies, and many had moderate to high risk of bias, partially mitigated by repeated sensitivity analyses excluding high-risk studies. Subgroup analyses based on small numbers of studies (e.g., CGM k = 2) should be considered exploratory only. The PGDM subgroup was too heterogeneous and underpowered for reliable conclusions; T1DM and T2DM are pathophysiologically distinct and should be separately enrolled in future RCTs. No study formally quantified equity outcomes stratified by sociodemographic characteristics, representing a critical evidence gap. Details on patient selection for remote care, including baseline complexity, comorbidities, and exclusion criteria, were frequently absent, limiting assessment of selection bias and whether remote care populations were systematically lower risk than comparator groups. In many clinical settings, remote care is offered preferentially to patients deemed suitable by prior clinical assessment, meaning the included populations may not be representative of the full spectrum of diabetes in pregnancy, particularly those with T1DM or insulin-dependent T2DM. Remote care encompasses a wide range of interventions, many multicomponent, making it difficult to isolate the effect of any individual element. Variation in multidisciplinary team composition, including differences in the intensity, structure, and content of care provided, represents an additional source of clinical heterogeneity and may have influenced observed outcomes independently of the remote care modality itself, such as GWG. Diagnostic criteria and timing thresholds for GDM varied across studies, potentially introducing further heterogeneity, although no studies included late GDM diagnoses. Finally, this review focused on the antepartum and intrapartum periods; long-term postpartum outcomes were not assessed, despite their relevance given the risk of T2DM and metabolic sequelae following GDM.^96^

Future research should extend beyond the immediate perinatal outcomes to examine long-term maternal and offspring health, including T2DM incidence and early growth and metabolic trajectories in children. Qualitative research should be used to explore experiences, preferences, and expectations of both patients and clinicians. Understanding these perspectives is vital for optimising uptake, adherence, and trust. Through pragmatic trial designs and implementation science frameworks, equity should be formally evaluated using sociodemographic outcomes, with attention to differential uptake, sustained engagement, and digital accessibility to ensure that remote care does not exacerbate hidden health inequalities. A network meta-analysis comparing the relative effectiveness of different remote care interventions would enable more refined comparisons. Additionally, further work is needed to examine how the mode of care influences clinical decision making; particularly as the varying rates of elective caesarean sections observed may reflect possible increased clinical caution or improved delivery planning. Comprehensive cost-effectiveness analyses accounting for downstream utilisation and digital infrastructure investment are also needed. Prior risk stratification should be undertaken before offering remote care to pregnant women with diabetes, to ensure that remote models are reserved for those with clinically appropriate complexity and adequate access to digital tools. These priorities align with the NHS 10-Year Health Plan, which identifies digitally enabled remote care as the standard for diabetes management.^97^

In summary, this systematic review and meta-analysis found that remote care for pregnant women with diabetes is largely comparable to usual care across key maternal and neonatal outcomes, with notable safety advantages including reduced emergency caesarean sections and NICU admissions in RCTs. Improvements in postpartum glycaemic control and GWG were observed in non-randomised studies, and patient satisfaction was consistently high. However, these findings are predominantly derived from populations with GDM managed in adequately resourced settings, where patients were selectively offered remote care based on clinical suitability; the evidence base for higher-complexity populations, including those with Type 1 diabetes or insulin-dependent Type 2 diabetes in pregnancy, remains insufficient to draw generalisable conclusions. Mixed findings regarding efficiency and timeliness were also observed. As remote care becomes increasingly embedded in maternity services, its adoption should be preceded by appropriate risk stratification to identify women for whom remote models are clinically suitable. Future research should prioritise long-term outcomes, higher-complexity diabetes subtypes, and consistent quality evaluation across all domains of care.

## Contributors

SS: Conceptualisation, Methodology, Data curation, Formal analysis, Investigation, Writing–original draft. ELE: Methodology, Data curation, Formal analysis, Writing–reviewing & editing. SPG: Methodology, Data curation, Formal analysis, Writing–reviewing & editing. GG: Methodology, Supervision, Writing–reviewing & editing. BH: Methodology, Supervision, Writing–reviewing & editing. SD: Supervision, Writing—reviewing & editing. NS: Methodology, Supervision, Writing—reviewing & editing. AM: Methodology, Supervision, Writing–reviewing & editing. AD: Conceptualisation, Methodology, Supervision, Project administration, Writing–reviewing & editing. ALN: Conceptualisation, Methodology, Formal analysis, Supervision, Project administration, Writing–reviewing & editing. SS, ELE, and SPG accessed and verified the data. All authors read and approved the final version of the manuscript.

## Data sharing statement

The data that support the findings of this study are based on previously published studies. The extracted dataset is available from the corresponding author upon reasonable request.

## Declaration of interests

SS, AD, and ALN are supported by the National Institute for Health and Care Research under the North West London Patient Safety Research Collaboration (NIHR NWL PSRC, Ref: NIHR204292), with infrastructure support from NIHR Imperial Biomedical Research Centre. ELE, GG, BH, AM, and ALN are also funded by the NIHR NWL Applied Research Collaboration (NIHR NWL ARC, Ref: NIHR200180). The views expressed in this publication are those of the authors and not necessarily those of the NIHR or the Department of Health and Social Care. BH also works for eConsult Health Ltd, a provider of online consultations for National Health Service primary, secondary, and urgent and emergency care. Other authors have no conflicts of interest to disclose.

## References

[1] Sweeting A., Hannah W., Backman H. (2024). Epidemiology and management of gestational diabetes. Lancet.

[2] Plows J.F., Stanley J.L., Baker P.N., Reynolds C.M., Vickers M.H. (2018). The pathophysiology of gestational diabetes mellitus. Int J Mol Sci.

[3] Zhang Y., Xiao C.-M., Zhang Y. (2021). Factors associated with gestational diabetes mellitus: a meta-analysis. J Diabetes Res.

[4] Roberto V., John R.T., Serdar H.U. (2010). Type 1 diabetes mellitus and pregnancy. Rev Obstet Gynecol.

[5] Dabelea D., Mayer-Davis E.J., Lamichhane A.P. (2008). Association of intrauterine exposure to maternal diabetes and obesity with type 2 diabetes in youth: the SEARCH Case-Control Study. Diabetes Care.

[6] Yang Y., Wu N. (2022). Gestational diabetes mellitus and preeclampsia: correlation and influencing factors. Front Cardiovasc Med.

[7] Mora-Ortiz M., Rivas-García L. (2024). Gestational diabetes mellitus: unveiling maternal health dynamics from pregnancy through postpartum perspectives. Open Research Europe.

[8] Sullivan S.D., Umans J.G., Ratner R. (2012). Gestational diabetes: implications for cardiovascular health. Curr Diab Rep.

[9] Ikomi A., Mannan S. (2022). The GooD pregnancy network: an alternative approach for gestational diabetes. BioMed.

[10] Griffin M.E., Coffey M., Johnson H. (2000). Universal vs. risk factor-based screening for gestational diabetes mellitus: detection rates, gestation at diagnosis and outcome. Diabet Med.

[11] Wolfe A. (2001).

[12] Campbell K., Greenfield G., Li E. (2023). The impact of virtual consultations on the quality of primary care: systematic review. J Med Internet Res.

[13] Neves A.L., Freise L., Laranjo L., Carter A.W., Darzi A., Mayer E. (2020). Impact of providing patients access to electronic health records on quality and safety of care: a systematic review and meta-analysis. BMJ Qual Saf.

[14] Neves A.L., Carter A.W., Freise L., Laranjo L., Darzi A., Mayer E.K. (2018). Impact of sharing electronic health records with patients on the quality and safety of care: a systematic review and narrative synthesis protocol. BMJ Open.

[15] Aldakhil R., Lammila-Escalera E., Hayhoe B., Majeed A., Greenfield G., Neves A.L. (2024). Impact of virtual consultations on quality of care in type 2 diabetes: a systematic review and narrative synthesis protocol. BMJ Open.

[16] Ravi S., Meyerowitz-Katz G., Yung C. (2025). Effect of virtual care in type 2 diabetes management – a systematic umbrella review of systematic reviews and meta-analysis. BMC Health Serv Res.

[17] Sousi S., Greenfield G., Hayhoe B.W.J. (2026). The impact of remote care on the quality of care of pregnant women with diabetes: a systematic review protocol. Syst Rev.

[18] Page M.J., McKenzie J.E., Bossuyt P.M. (2021). The PRISMA 2020 statement: an updated guideline for reporting systematic reviews. PLoS Med.

[19] Covidence Systematic Review Software, Veritas Health Innovation, Melbourne, Australia. http://www.covidence.org.

[20] McHugh M.L. (2012). Interrater reliability: the kappa statistic. Biochem Med.

[21] Chi K.-Y., Li M.-Y., Chen C., Kang E. (2023). Ten circumstances and solutions for finding the sample mean and standard deviation for meta-analysis. Syst Rev.

[22] Sterne J.A., Hernán M.A., Reeves B.C. (2016). ROBINS-I: a tool for assessing risk of bias in non-randomised studies of interventions. BMJ.

[23] Higgins J.P.T., Altman D.G., Gotzsche P.C. (2011). The Cochrane Collaboration's tool for assessing risk of bias in randomised trials. BMJ.

[24] R Core Team (2024). https://www.R-project.org/.

[25] Al-ofi E.A., Mosli H.H., Ghamri K.A., Ghazali S.M. (2019). Management of postprandial hyperglycaemia and weight gain in women with gestational diabetes mellitus using a novel telemonitoring system. J Int Med Res.

[26] Amylidi-Mohr S., Zennaro G., Schneider S., Raio L., Mosimann B., Surbek D. (2025). Continuous glucose monitoring in the management of gestational diabetes in Switzerland (DipGluMo): an open-label, single-centre, randomised, controlled trial. Lancet Diabetes Endocrinol.

[27] Borgen I., Småstuen M.C., Jacobsen A.F. (2019). Effect of the pregnant+ smartphone application in women with gestational diabetes mellitus: a randomised controlled trial in Norway. BMJ Open.

[28] Bromuri S., Puricel S., Schumann R., Krampf J., Ruiz J., Schumacher M. (2016). An expert Personal Health System to monitor patients affected by Gestational Diabetes Mellitus: a feasibility study. J Ambient Intell Smart Environ.

[29] Carral F., Ayala M., Fernández J.J. (2015). Web-based telemedicine system is useful for monitoring glucose control in pregnant women with diabetes. Diabetes Technol Ther.

[30] Chatzakis C., Floros D., Liberis A. (2022). STORK: collaborative online monitoring of pregnancies complicated with gestational diabetes mellitus. Healthcare.

[31] Clark A., Jung E., Prusky C., Shah B.R., Halperin I.J. (2023). An evaluation of virtual care for gestational diabetes using the quadruple aim framework: assessment of patient and provider experience, cost, and clinical outcomes. Can J Diabetes.

[32] Dalfrà M.G., Nicolucci A., Lapolla A. (2009). The effect of telemedicine on outcome and quality of life in pregnant women with diabetes. J Telemed Telecare.

[33] Gaulier S., Sonnet E., Thuillier P., Crouzeix G., Bounceur A., Kerlan V. (2017). Évaluation d’un programme de suivi de patientes avec un diabète gestationnel par télémédecine : expérience brestoise. Médecine des Maladies Métaboliques.

[34] Given J.E., Bunting B.P., O'Kane M.J., Dunne F., Coates V.E. (2015). Tele-Mum: a feasibility study for a randomized controlled trial exploring the potential for telemedicine in the diabetes care of those with gestational diabetes. Diabetes Technol Ther.

[35] Guo H., Zhang Y., Li P., Zhou P., Chen L.-M., Li S.-Y. (2019). Evaluating the effects of mobile health intervention on weight management, glycemic control and pregnancy outcomes in patients with gestational diabetes mellitus. J Endocrinol Investig.

[36] Homko C.J., Santamore W.P., Whiteman V. (2007). Use of an internet-based telemedicine system to manage underserved women with gestational diabetes mellitus. Diabetes Technol Ther.

[37] Homko C.J., Deeb L.C., Rohrbacher K. (2012). Impact of a telemedicine system with automated reminders on outcomes in women with gestational diabetes mellitus. Diabetes Technol Ther.

[38] Huang B., Zhai H. (2025). A retrospective study of WeChat app-based health management for patients with gestational diabetes mellitus. J Obstet Gynaecol (Lahore).

[39] Hyams E., Brackenridge A., White S.L. (2024). Impact of the use of a smartphone-based blood glucose management system within a maternal diabetes service. Practical Diabetes.

[40] Kantorowska A., Cohen K., Oberlander M. (2023). Remote patient monitoring for management of diabetes mellitus in pregnancy is associated with improved maternal and neonatal outcomes. Am J Obstet Gynecol.

[41] Laurie J.G., Wilkinson S.A., Griffin A., McIntyre H.D. (2023). GDM care re-imagined: maternal and neonatal outcomes following a major model of care change for gestational diabetes mellitus at a large metropolitan hospital. Aust N Z J Obstet Gynaecol.

[42] Lemelin A., Paré G., Bernard S., Godbout A. (2020). Demonstrated cost-effectiveness of a telehomecare program for gestational diabetes mellitus management. Diabetes Technol Ther.

[43] Leyris Z., Bidan L., Puel Q. (2024). Improving gestational diabetes care: mobile glucose monitoring to reduce complications. Ann Endocrinol.

[44] Mackillop L., Hirst J.E., Bartlett K.J. (2018). Comparing the efficacy of a mobile phone-based blood glucose management system with standard clinic care in women with gestational diabetes: randomized controlled trial. JMIR mHealth uHealth.

[45] Mclennan N., Lindsay R., Saravanan P. (2024). Impact of COVID-19 on gestational diabetes pregnancy outcomes in the UK: a multicentre retrospective cohort study. BJOG.

[46] Miremberg H., Ben-Ari T., Betzer T. (2018). The impact of a daily smartphone-based feedback system among women with gestational diabetes on compliance, glycemic control, satisfaction, and pregnancy outcome: a randomized controlled trial. Am J Obstet Gynecol.

[47] Montori S., Lugli F., Monesi M. (2024). Telemedicine in the treatment of gestational diabetes: an observational cohort study on pregnancy outcomes and maternal satisfaction. Diabet Med.

[48] Munda A., Mlinaric Z., Jakin P.A., Lunder M., Pongrac Barlovic D. (2023). Effectiveness of a comprehensive telemedicine intervention replacing standard care in gestational diabetes: a randomized controlled trial. Acta Diabetol.

[49] Peleg M., Shahar Y., Quaglini S. (2017). Assessment of a personalized and distributed patient guidance system. Int J Med Inform.

[50] Pérez-Ferre N., Galindo M., Fernández M.D. (2010). The outcomes of gestational diabetes mellitus after a telecare approach are not inferior to traditional outpatient clinic visits. Int J Endocrinol.

[51] Poncelet C., Bouamoud L., Michel P., Campinos C. (2024). Monitoring gestational diabetes mellitus patients with myDiabby Healthcare® smartphone application vs classical diary. Results from the non-inferiority TELESUR-GDM study. Diabetes Metab.

[52] Rasekaba T.M., Furler J., Young D. (2018). Using technology to support care in gestational diabetes mellitus: quantitative outcomes of an exploratory randomised control trial of adjunct telemedicine for gestational diabetes mellitus (TeleGDM). Diabetes Res Clin Pract.

[53] Shashikumar A., Oyston C., Okesene-Gafa K., Apaapa-Timu T.H., Wilson J. (2022). Teleclinics for the management of diabetes in pregnancy during COVID-19—maternal satisfaction and pregnancy outcomes. N Z Med J.

[54] Sung Y.-S., Zhang D., Eswaran H., Lowery C.L. (2021). Evaluation of a telemedicine program managing high-risk pregnant women with pre-existing diabetes in Arkansas's Medicaid program. Semin Perinatol.

[55] Syrop C.H., Wernimont S.A., Fleener D.K., Kardos J.M., Rubenstein L.M., Andrews J.I. (2021). Redesigned care delivery for insulin-requiring diabetes in pregnancy improves perinatal glycemic control while reducing neonatal intensive care admissions, length of stay, and costs. J Womens Health.

[56] Tian Y., Zhang S., Huang F., Ma L. (2021). Comparing the efficacies of telemedicine and standard prenatal care on blood glucose control in women with gestational diabetes mellitus: randomized controlled trial. JMIR mHealth uHealth.

[57] Wang Q., Zhang K., Zhang X. (2025). WeChat mini-program, a preliminary applied study of the gestational blood glucose management model for pregnant women with gestational diabetes mellitus. Diabetes Res Clin Pract.

[58] Wei Q., Sun Z., Yang Y., Yu H., Ding H., Wang S. (2016). Effect of a CGMS and SMBG on maternal and neonatal outcomes in gestational diabetes mellitus: a randomized controlled trial. Sci Rep.

[59] Zhuo Y., Pan Y., Lin K. (2022). Effectiveness of clinical pharmacist-led smartphone application on medication adherence, insulin injection technique and glycemic control for women with gestational diabetes receiving multiple daily insulin injection: a randomized clinical trial. Prim Care Diabetes.

[60] Wen J., Zhou L., Hu H. (2025). Evaluation of a lifestyle intervention for women with gestational diabetes mellitus based on the medical internet of things: a randomized controlled trial with mid-sample verification. Hypertens Pregnancy.

[61] Yew T.W., Chi C., Chan S.-Y. (2021). A randomized controlled trial to evaluate the effects of a smartphone application–based lifestyle coaching program on gestational weight gain, glycemic control, and maternal and neonatal outcomes in women with gestational diabetes mellitus: the SMART-GDM study. Diabetes Care.

[62] Laurie J.G., Wilkinson S.A., Mcintyre H.D., Snoswell C. (2023). Gestational diabetes mellitus care re-imagined – a cost-minimisation analysis: cost savings from a tertiary hospital, using a novel, digital-based gestational diabetes management model. Aust N Z J Obstet Gynaecol.

[63] Kytö M., Hotta S., Niinistö S. (2024). Periodic mobile application (eMOM) with self-tracking of glucose and lifestyle improves treatment of diet-controlled gestational diabetes without human guidance: a randomized controlled trial. Am J Obstet Gynecol.

[64] Fincher K., Fiocchi C., Odom J., Stancil M., White H., Schellinger M. (2025). Pregestational diabetes intervention pilot study with diabetes self-management education and support and cellular-enabled glucose meter. Diabetes Spectr.

[65] Dolatabadi S., Yamamoto J.M., Brennand E.A., Donovan L.E., Benham J.L. (2025). Virtual vs in-person care in gestational diabetes management: a retrospective cohort analysis. Can J Diabetes.

[66] Coveney C., Callaghan S., Rutter E. (2026). Implementation of a gestational diabetes virtual care clinic: a before–after comparative study. Diabetes Ther.

[67] Cichocka E., Gumprecht J. (2024). The impact of telemedicine during the COVID-19 pandemic on diabetes management and pregnancy outcomes in women with gestational diabetes mellitus (GDM). J Clin Med.

[68] Berezowsky A., Melamed N., Murray-Davis B. (2024). Impact of antenatal care modifications on gestational diabetes outcomes during the COVID-19 pandemic. Can J Diabetes.

[69] Bartholomew M.L., Soules K., Church K. (2015). Managing diabetes in pregnancy using cell phone/internet technology. Clin Diabetes.

[70] McMicking J., Lam A.Y.R., Lim W., Tan A.P.L.-K., Pasupathy P.D. (2021). Epidemiology and classification of diabetes in pregnancy. Gloal Librf Womens Med.

[71] London: National Institute for Health and Care Excellence (NICE) (2020). Diabetes in pregnancy: management from preconception to the postnatal period. (NICE Guideline, No. 3). https://www.ncbi.nlm.nih.gov/books/NBK555331/.

[72] Lloyd M., Morton J., Teede H. (2023). Long-term cost-effectiveness of implementing a lifestyle intervention during pregnancy to reduce the incidence of gestational diabetes and type 2 diabetes. Diabetologia.

[73] Oliveira A.P.V.D., Zaccara T.A., Bernardi M.D.C. (2025). Risk factors and primary indications for cesarean section in women with gestational diabetes mellitus according to Robson classification. Clinics.

[74] Khin T.N., Ang W.W., Lau Y. (2025). The effect of smartphone application–based self-management interventions compared to face-to-face diabetic interventions for pregnant women with gestational diabetes mellitus: a meta-analysis. J Diabetes Res.

[75] Overdijkink S.B., Velu A.V., Rosman A.N., van Beukering M.D., Kok M., Steegers-Theunissen R.P. (2018). The usability and effectiveness of mobile health technology–based lifestyle and medical intervention apps supporting health care during pregnancy: systematic review. JMIR mHealth uHealth.

[76] Halligan J., Whelan M.E., Roberts N., Farmer A.J. (2021). Reducing weight and BMI following gestational diabetes: a systematic review and meta-analysis of digital and telemedicine interventions. BMJ Open Diabetes Res Care.

[77] Raman P., Shepherd E., Dowswell T., Middleton P., Crowther C.A. (2017). Different methods and settings for glucose monitoring for gestational diabetes during pregnancy. Cochrane Database Syst Rev.

[78] Pires-Menard A., Flatley C., Kumar S. (2021). Severe neonatal outcomes associated with emergency cesarean section at term. J Matern Fetal Neonatal Med.

[79] Reader D., Splett P., Gunderson E.P. (2006). Impact of gestational diabetes mellitus nutrition practice guidelines implemented by registered dietitians on pregnancy outcomes. J Am Diet Assoc.

[80] Wilkinson S.A., Willcox J.C. (2022). Is ‘technology before the end-user’ the new ‘cart before the horse’? When digital delivery is only part of the solution. JBI Evid Implement.

[81] Palmer K.R., Tanner M., Davies-Tuck M. (2021). Widespread implementation of a low-cost telehealth service in the delivery of antenatal care during the COVID-19 pandemic: an interrupted time-series analysis. Lancet.

[82] de Jersey S., Keramat S.A., Chang A. (2024). A cost-effectiveness evaluation of a dietitian-delivered telephone coaching program during pregnancy for preventing gestational diabetes mellitus. Cost Eff Resour Allocation.

[83] Lappe V., Greiner G.G., Linnenkamp U. (2023). Gestational diabetes in Germany—Prevalence, trend during the past decade and utilization of follow-up care: an observational study. Sci Rep.

[84] Luo R., Fell D.B., Corsi D.J., Taljaard M., Wen S.W., Walker M.C. (2024). Temporal trends in gestational diabetes mellitus and associated risk factors in Ontario, Canada, 2012–2020: a population-based cohort study. J Obstet Gynaecol Can.

[85] Zhou T., Du S., Sun D. (2022). Prevalence and trends in gestational diabetes mellitus among women in the United States, 2006–2017: a population-based study. Front Endocrinol.

[86] Flack J.R., Ross G.P., Ho S., McELDUFF A. (2010). Recommended changes to diagnostic criteria for gestational diabetes: impact on workload. Aust N Z J Obstet Gynaecol.

[87] Martis R., Brown J., McAra-Couper J., Crowther C.A. (2018). Enablers and barriers for women with gestational diabetes mellitus to achieve optimal glycaemic control – a qualitative study using the theoretical domains framework. BMC Pregnancy Childbirth.

[88] Sousi S., Greenfield G., Hayhoe B.W.J. (2025).

[89] Wang F., Yu K., Li Z. (2020). Effectiveness of telemedicine for glucose control and pregnancy outcomes in gestational diabetes mellitus: systematic review and meta-analysis. 中华健康管理学杂志.

[90] Hangaard S., Laursen S.H., Udsen F.W., Vestergaard P., Hejlesen O. (2020). Studies in health technology and informatics.

[91] Huang N., Chen S., Zhou Y. (2019). Efficacy and safety of telemedicine for blood glucose and pregnancy outcomes in gestational diabetes mellitus: a systematic review. Chin J Evidence-Based Med.

[92] Xie W., Dai P., Qin Y., Wu M., Yang B., Yu X. (2020). Effectiveness of telemedicine for pregnant women with gestational diabetes mellitus: an updated meta-analysis of 32 randomized controlled trials with trial sequential analysis. BMC Pregnancy Childbirth.

[93] Sockalingam S., Kirvan A., Pereira C. (2021). https://home.liebertpub.com/tmj.

[94] Shenoy A. (2021). Patient safety from the perspective of quality management frameworks: a review. Patient Saf Surg.

[95] Agency for Healthcare Research and Quality, Rockville MD Six domains of healthcare quality. 2025. https://www.ahrq.gov/talkingquality/measures/six-domains.html.

[96] Phaloprakarn C., Tangjitgamol S. (2022). Glucose levels during gestational diabetes pregnancy and the risk of developing postpartum diabetes or prediabetes. BMC Pregnancy Childbirth.

[97] Department of Health and Social Care (2025). 10 year health plan for England: fit for the future. https://assets.publishing.service.gov.uk/media/6866387fe6557c544c74db7a/fit-for-the-future-10-year-health-plan-for-england.pdf.

