# Supplementary material for: The impact of remote care on the quality of care of pregnant women with diabetes: a systematic review and meta-analysis
Source: eClinicalMedicine. 2026 Aug 18;99:104147. doi: 10.1016/j.eclinm.2026.104147 (PMC13507814; doi:10.1016/j.eclinm.2026.104147)
Supplement: Supplementary file 1 [file mmc1.docx]

**“The Impact of Remote Care on the Quality of Care of Pregnant Women with Diabetes: A Systematic Review and Meta-Analysis”**

**Supplementary Material**

[Appendix A – Search Strategy by Database	2](#_Toc235794985)

[Appendix B – Risk of Bias	8](#_Toc235794986)

[Appendix C – Efficiency Results	10](#_Toc235794987)

[Appendix D – Sensitivity Analysis By Diabetes Type	12](#_Toc235794988)

[Appendix E – Sensitivity Analysis By Remote Care Type	14](#_Toc235794989)

[Appendix F – Sensitivity Analysis By Risk of Bias	19](#_Toc235794990)

[Appendix G – Forest Plots	21](#_Toc235794991)

[Appendix H – Funnel Plots	28](#_Toc235794992)

# Appendix A – Search Strategy by Database

**Table A1** Inclusion and Exclusion Criteria of the Population, Intervention, Comparison, Outcomes, and Study (PICOS) framework

|  | **Inclusion Criteria** | **Exclusion Criteria** |
| --- | --- | --- |
| **Population** | - Pregnant women with pre-existing diabetes mellitus (type 1 or type 2) - Pregnant women with gestational diabetes mellitus | - Non-pregnant women with diabetes mellitus (type 1 or type 2) - Children (age < 16) or men with diabetes mellitus (type 1 or type 2) - Post-partum women |
| **Intervention** | Remote care for clinical management of diabetes in pregnancy, this can include:   - Telemedicine including virtual / remote consultations (telephone, video, online, chat based, hybrid) - Remote monitoring applications and systems, such as mobile applications (mHealth) and web-based systems - Remote monitoring devices such as CGMs - Virtual wards | - Teletherapy support interventions - Virtual reality interventions - Digital tools aimed for post-pregnancy - Digital tools aiding in diagnosis and / or screening of pregnant women for gestational diabetes mellitus - Digital interventions that are lifestyle, exercise, and education purposed only |
| **Comparator** | Face-to-face monitoring  Usual standard of care | Non usual care or in-person monitoring  Non-comparator (single arm) studies |
| **Outcomes** | IoM Quality of Care Domains:   - Effectiveness - Safety - Efficiency - Timeliness - Equity - Patient-centredness | N/A |
| **Study type** | Studies with empirical data, original research: randomised control trials, non-randomised control trials, cohort studies, retrospective studies, cross-sectional studies, case-control studies | Reviews, abstracts, editorials, book chapters, commentaries, case reports, non-full-text studies, protocols, conference proceedings |

| **Ovid MEDLINE(R) ALL <1946 to April 25, 2025>** | | | |  |
| --- | --- | --- | --- | --- |
| **Search Strategy:** | | | |  |
| **1** | exp Telemedicine/ or telemedicine·mp· | 66,063 |  |  |
| **2** | exp Remote Consultation/ | 6,164 |  |  |
| **3** | ((online or phone* or remote* or tele* or video* or virtual*) adj2 (appointment* or care or consult* or hospital* or monitor* or visit* or ward*))·mp· | 47,407 |  |  |
| **4** | (digital health or dhealth or d-health or home monitor* or mobile health or mHealth or m-Health or smartphone* app* or mobile* or long distance consultation* or teleconsult* or tele-consult* or telemonitor* or tele-monitor* or telehealth* or tele health* or tele-health* or telemetry or tele-homecare or telehomecare or remote surveillance or remotely delivered or tele nursing or telenursing)·mp· | 217,232 |  |  |
| **5** | 1 or 2 or 3 or 4 | 277,597 |  |  |
| **6** | exp Diabetes, Gestational/ or gestational diabetes·mp· | 29,917 |  |  |
| **7** | exp Pregnancy/ and exp Diabetes Mellitus/ | 30,894 |  |  |
| **8** | (pregnan* adj3 (gly* or diabet* or DM or GDM))·mp· | 23,141 |  |  |
| **9** | (pregestational diabetes or pre-gestational diabet* or maternal diabet*)·mp· | 3,838 |  |  |
| **10** | exp Pregnancy in Diabetics/ | 14,244 |  |  |
| **11** | 6 or 7 or 8 or 9 or 10 | 49,161 |  |  |
| **12** | (((experience* or satisfaction) adj4 (patient* or consumer* or client* or survey* or questionnaire*)) or PREM* or patient-reported experience measure* or patient-cent?red* or person-cent?red*)·mp· | 888,576 |  |  |
| **13** | exp Patient Satisfaction/ | 106,407 |  |  |
| **14** | 12 or 13 | 897,871 |  |  |
| **15** | exp Treatment Outcome/ or ((health or clinical* or treatment*) adj3 (outcome* or effective* or efficacy))·mp· | 2,220,251 |  |  |
| **16** | ((patient adj3 (safety or harm)) or misdiagnos* or safety manag* or (accident* adj2 prevent*) or error* or medication reconcil* or near miss*)·mp· | 644,875 |  |  |
| **17** | exp patient harm/ or exp patient safety/ or exp Diagnostic Errors/ | 152,444 |  |  |
| **18** | 16 or 17 | 723,774 |  |  |
| **19** | (efficien* or economic* or cost* or expenditure* or charge* or (number adj3 appointment*) or (number adj3 admission*) or (number adj3 consultation*))·mp· | 3,253,601 |  |  |
| **20** | (wait* list* or wait* time* or timeliness)·mp· | 50,527 |  |  |
| **21** | exp Time-to-Treatment/ or exp Waiting Lists/ | 25,908 |  |  |
| **22** | 20 or 21 | 61,098 |  |  |
| **23** | ((health* or health care or access) adj3 (equity or disparit* or inequit* or inequalit* or equality or gap))·mp· | 115,914 |  |  |
| **24** | exp Health Equity/ | 5,219 |  |  |
| **25** | 23 or 24 | 115,914 |  |  |
| **26** | 14 or 15 or 18 or 19 or 22 or 25 | 6,626,078 |  |  |
| **27** | 5 and 11 and 26 | 248 |  |  |
| **28** | limit 27 to yr="2005 -Current" | 235 |  |  |

| **Embase Classic + Embase <1947 to 2025 April 25>** | | | |  |
| --- | --- | --- | --- | --- |
| **Search Strategy:** | | | |  |
| **1** | telemedicine·mp· or exp telemedicine/ | 91,976 |  |  |
| **2** | exp teleconsultation/ | 18,130 |  |  |
| **3** | ((online or phone* or remote* or tele* or video* or virtual*) adj2 (appointment* or care or consult* or hospital* or monitor* or visit* or ward*))·mp· | 68,086 |  |  |
| **4** | (digital health or dhealth or d-health or home monitor* or mobile health or mHealth or m-Health or smartphone* app* or mobile* or long distance consultation* or teleconsult* or tele-consult* or telemonitor* or tele-monitor* or telehealth* or tele health* or tele-health* or telemetry or tele-homecare or telehomecare or remote surveillance or remotely delivered or tele nursing or telenursing)·mp· | 328,258 |  |  |
| **5** | 1 or 2 or 3 or 4 | 410,107 |  |  |
| **6** | exp gestational diabetes/ or exp maternal diabetes mellitus/ or gestational diabetes·mp· | 68,712 |  |  |
| **7** | (pregnan* adj3 (gly* or diabet* or DM or GDM))·mp· | 27,607 |  |  |
| **8** | (pregestational diabetes or pre-gestational diabet* or maternal diabet*)·mp· | 11,173 |  |  |
| **9** | exp pregnancy in diabetics/ | 10,475 |  |  |
| **10** | 6 or 7 or 8 or 9 | 80,023 |  |  |
| **11** | (((experience* or satisfaction) adj4 (patient* or consumer* or client* or survey* or questionnaire*)) or PREM* or patient-reported experience measure* or patient-cent?red* or person-cent?red*)·mp· | 1,347,837 |  |  |
| **12** | exp Patient Satisfaction/ | 187,969 |  |  |
| **13** | 11 or 12 | 1,347,837 |  |  |
| **14** | exp Treatment Outcome/ or ((health or clinical* or treatment*) adj3 (outcome* or effective* or efficacy))·mp· | 3,910,398 |  |  |
| **15** | ((patient adj3 (safety or harm)) or misdiagnos* or safety manag* or (accident* adj2 prevent*) or error* or medication reconcil* or near miss*)·mp· | 1,151,441 |  |  |
| **16** | exp patient harm/ or exp patient safety/ or exp Diagnostic Errors/ | 329,931 |  |  |
| **17** | 15 or 16 | 1,224,965 |  |  |
| **18** | (efficien* or economic* or cost* or expenditure* or charge* or (number adj3 appointment*) or (number adj3 admission*) or (number adj3 consultation*))·mp· | 3,210,533 |  |  |
| **19** | (wait* list* or wait* time* or timeliness)·mp· | 69,987 |  |  |
| **20** | exp Time-to-Treatment/ or exp Waiting Lists/ | 345,756 |  |  |
| **21** | 19 or 20 | 397,386 |  |  |
| **22** | ((health* or health care or access) adj3 (equity or disparit* or inequit* or inequalit* or equality or gap))·mp· | 145,091 |  |  |
| **23** | exp health equity/ | 15,382 |  |  |
| **24** | 22 or 23 | 145,091 |  |  |
| **25** | 13 or 14 or 17 or 18 or 21 or 24 | 8,991,945 |  |  |
| **26** | 5 and 10 and 25 | 540 |  |  |
| **27** | limit 26 to yr="2005 -Current" | 519 |  |  |

| **Database: Global Health <1973 to 2025 Week 16>** | | | |  |
| --- | --- | --- | --- | --- |
| **Search Strategy:** | | | |  |
| **1** | telemedicine·mp· | 6,610 |  |  |
| **2** | ((online or phone* or remote* or tele* or video* or virtual*) adj2 (appointment* or care or consult* or hospital* or monitor* or visit* or ward*))·mp· | 5,492 |  |  |
| **3** | (digital health or dhealth or d-health or home monitor* or mobile health or mHealth or m-Health or smartphone* app* or mobile* or long distance consultation* or teleconsult* or tele-consult* or telemonitor* or tele-monitor* or telehealth* or tele health* or tele-health* or telemetry or tele-homecare or telehomecare or remote surveillance or remotely delivered or tele nursing or telenursing)·mp· | 46,950 |  |  |
| **4** | 1 or 2 or 3 | 53,762 |  |  |
| **5** | exp gestational diabetes/ or gestational diabetes·mp· | 8,784 |  |  |
| **6** | (pregnancy and (diabetes mellitus or diabetes))·mp· | 12,821 |  |  |
| **7** | (pregnan* adj3 (gly* or diabet* or DM or GDM))·mp· | 4,264 |  |  |
| **8** | (pregestational diabetes or pre-gestational diabet* or maternal diabet*)·mp· | 854 |  |  |
| **9** | pregnancy in diabetics·mp· | 7 |  |  |
| **10** | 5 or 6 or 7 or 8 or 9 | 14,291 |  |  |
| **11** | (((experience* or satisfaction) adj4 (patient* or consumer* or client* or survey* or questionnaire*)) or PREM* or patient-reported experience measure* or patient-cent?red* or person-cent?red*)·mp· | 111,905 |  |  |
| **12** | patient satisfaction·mp· | 4,028 |  |  |
| **13** | 11 or 12 | 111,905 |  |  |
| **14** | exp Treatment Outcome/ or ((health or clinical* or treatment*) adj3 (outcome* or effective* or efficacy))·mp· | 211,235 |  |  |
| **15** | ((patient adj3 (safety or harm)) or misdiagnos* or safety manag* or (accident* adj2 prevent*) or error* or medication reconcil* or near miss*)·mp· | 58,933 |  |  |
| **16** | patient harm·mp· or exp patient safety/ or exp Diagnostic Errors/ | 203 |  |  |
| **17** | 15 or 16 | 58,933 |  |  |
| **18** | (efficiency or economic* or cost* or expenditure* or charge* or (number adj3 appointment*) or (number adj3 admission*) or (number adj3 consultation*))·mp· | 602,968 |  |  |
| **19** | (wait* list* or wait* time* or timeliness)·mp· | 8,477 |  |  |
| **20** | (time-to-treatment or "time to treatment" or waiting list)·mp· | 4,207 |  |  |
| **21** | 19 or 20 | 11,540 |  |  |
| **22** | ((health* or health care or access) adj3 (equity or disparit* or inequit* or inequalit* or equality or gap))·mp· | 41,737 |  |  |
| **23** | health equity·mp· | 4,561 |  |  |
| **24** | 22 or 23 | 41,737 |  |  |
| **25** | 13 or 14 or 17 or 18 or 21 or 24 | 943,712 |  |  |
| **26** | 4 and 10 and 25 | 44 |  |  |
| **27** | limit 26 to yr="2005 -Current" | 43 |  |  |

| **Maternity & Infant Care Database (MIDIRS) <1971 to April 22, 2025>** | | | |  |
| --- | --- | --- | --- | --- |
| **Search Strategy:** | | | |  |
| **1** | ((online or phone* or remote* or tele* or video* or virtual*) adj2 (appointment* or care or consult* or hospital* or monitor* or visit* or ward*))·mp· | 692 |  |  |
| **2** | (digital health or dhealth or d-health or home monitor* or mobile health or mHealth or m-Health or smartphone* app* or mobile* or long distance consultation* or teleconsult* or tele-consult* or telemonitor* or tele-monitor* or telehealth* or tele health* or tele-health* or telemedicine or tele-medicine or tele medicine or telemetry or tele-homecare or telehomecare or remote surveillance or remotely delivered or tele nursing or telenursing)·mp· | 1,871 |  |  |
| **3** | 1 or 2 | 2,302 |  |  |
| **4** | (pregnan* adj3 (gly* or diabet* or DM or GDM))·mp· | 3,737 |  |  |
| **5** | (pregestational diabetes or pre-gestational diabet* or maternal diabet*)·mp· | 1,104 |  |  |
| **6** | gestational diabet*·mp· | 7,211 |  |  |
| **7** | (pregnancy and diabetes mellitus)·mp· | 5,050 |  |  |
| **8** | 4 or 5 or 6 or 7 | 10,025 |  |  |
| **9** | (((experience* or satisfaction) adj4 (patient* or consumer* or client* or survey* or questionnaire*)) or PREM* or patient-reported experience measure* or patient-cent?red* or person-cent?red*)·mp· | 33,207 |  |  |
| **10** | ((health or clinical* or treatment*) adj3 (outcome* or effective* or efficacy))·mp· | 15,837 |  |  |
| **11** | ((patient adj3 (safety or harm)) or misdiagnos* or safety manag* or (accident* adj2 prevent*) or error* or diagnostic error* or medication reconcil* or near miss*)·mp· | 4,989 |  |  |
| **12** | (efficien* or economic* or cost* or expenditure* or charge* or (number adj3 appointment*) or (number adj3 admission*) or (number adj3 consultation*))·mp· | 20,462 |  |  |
| **13** | (wait* list* or wait* time* or timeliness or time-to-treatment or "time to treatment")·mp· | 712 |  |  |
| **14** | ((health* or health care or access) adj3 (equity or disparit* or inequit* or inequalit* or equality or gap))·mp· | 3,380 |  |  |
| **15** | 9 or 10 or 11 or 12 or 13 or 14 | 70,109 |  |  |
| **16** | 3 and 8 and 15 | 42 |  |  |
| **17** | limit 16 to yr="2005 -Current" | 39 |  |  |

| **Scopus** | | | |  |
| --- | --- | --- | --- | --- |
| **Search String** | | | |  |
| **1** | TITLE-ABS-KEY ( telemedicine OR teleconsultation OR ( (online OR phone* OR remote* OR tele* OR video* OR virtual* ) W/2 ( appointment* OR care OR consult* OR hospital* OR monitor* OR visit* OR ward* ) ) OR "digital health" OR dhealth OR d-health OR "home monitor*" OR "mobile health" OR mhealth OR m-health OR "long distance consultation*" OR teleconsult* OR tele-consult* OR telemonitor* OR tele-monitor* OR telehealth* OR "tele health*" OR tele-health* OR telemetry OR tele-homecare OR telehomecare OR "remote surveillance" OR "remotely delivered" OR "tele nursing" OR telenursing OR "self management" OR "self-management" OR "smartphone* app*" OR mobile* ) | 1,387,265 |  |  |
| **2** | TITLE-ABS-KEY ( "gestational diabetes" OR "maternal diabetes" OR ( pregnan* W/3 ( gly* OR diabet* OR dm OR gdm )) OR "pregestational diabetes" OR "pre-gestational diabetes" ) | 65,198 |  |  |
| **3** | TITLE-ABS-KEY ( ( ( experience* OR satisfaction ) W/4 ( patient* OR consumer* OR client* OR survey* OR questionnaire* ) ) OR prem* OR "patient-reported experience measure*" OR patient-cent*red* OR person-cent*red* ) | 1,540,397 |  |  |
| **4** | TITLE-ABS-KEY((health OR clinical* OR treatment* ) W/3 (outcome* OR effective* OR efficacy )) | 3,129,424 |  |  |
| **5** | TITLE-ABS-KEY((patient W/3 (safety OR harm )) OR misdiagnos* OR "safety manag*" OR (accident* W/2 prevent* ) OR error* OR "medication reconcil*" OR "near miss*" ) | 3,170,821 |  |  |
| **6** | TITLE-ABS-KEY ( efficiency OR economic* OR cost* OR expenditure* OR charge* OR ( number W/3 appointment* ) OR ( number W/3 admission* ) OR ( number W/3 consultation* ) ) | 11,347,188 |  |  |
| **7** | TITLE-ABS-KEY ( "wait* list*" OR "wait* time*" OR timeliness OR time-to-treatment OR "time to treatment" ) | 139,970 |  |  |
| **8** | TITLE-ABS-KEY ( ( health* OR "health care" OR access ) W/3 ( equity OR disparit* OR inequit* OR inequalit* OR equality OR gap ) ) | 177,419 |  |  |
| **9** | 3 OR 4 OR 5 OR 6 OR 7 OR 8 | 18,053,124 |  |  |
| **10** | 1 AND 2 AND 9 | 552 |  |  |
| **11** | 10 AND PUBYEAR > 2004 | 519 |  |  |

| **Cochrane** | |  | | |  |
| --- | --- | --- | --- | --- | --- |
| **1** | ([mh telemedicine] OR [mh "remote consultation"] OR telemedicine OR telehealth OR "tele health" OR telemonitor* OR tele-monitor* OR teleconsult* OR tele-consult* OR telecare OR telenursing OR telemetry OR telehomecare OR "tele-homecare" OR (tele NEXT consult*) OR (tele NEXT monitor*) OR "remote surveillance" OR "remotely delivered" OR (virtual* NEAR/2 (consult* OR appointment* OR care OR hospital* OR monitor* OR visit* OR ward*)) OR (remote* NEAR/2 (consult* OR care OR hospital* OR monitor* OR visit* OR ward*)) OR (phone* NEAR/2 (consult* OR appointment* OR visit*)) OR (video* NEAR/2 (consult* OR appointment* OR visit*)) OR "digital health" OR dhealth OR d-health OR home monitor* OR "mobile health" OR mHealth OR m-health OR long distance consultation* OR "self management" OR "self-management" OR smartphone* app* OR mobile*) | | 58,593 |  |  |
| **2** | ([mh "diabetes, gestational"] OR ([mh pregnancy] AND [mh "diabetes mellitus"]) OR [mh "pregnancy in diabetics"] OR "gestational diabetes" OR GDM OR "maternal diabetes" OR "pregestational diabetes" OR "pre-gestational diabetes" OR (pregnan* NEAR/3 (diabet* OR DM OR GDM OR gly*)) OR "pregnancy-induced diabetes" OR "pregnancy induced diabetes" OR "diabetes during pregnancy" OR "diabetes in pregnancy" OR "diabetic pregnancy" OR ("pregnancy in" NEXT diabet*) OR (pregnan* NEAR/2 diabet*) OR (pregnan* NEAR/2 DM) OR (pregnan* NEAR/2 gly*) OR "woman DM") | | 5,395 |  |  |
| **3** | (((experience* OR satisfaction) NEAR/4 (patient* OR consumer* OR client* OR survey* OR questionnaire*)) OR PREM* OR ("patient-reported experience" NEXT measure*) OR patient-cent?red* OR person-cent?red*) OR [mh "Patient Satisfaction"] | | 151,329 |  |  |
| **4** | [mh "Treatment Outcome"] OR ((health OR clinical* OR treatment*) NEAR/3 (outcome* OR effective* OR efficacy)) | | 457,536 |  |  |
| **5** | ((patient NEAR/3 (safety OR harm)) OR misdiagnos* OR ("safety" NEXT manag*) OR (accident* NEAR/2 prevent*) OR error* OR ("medication" NEXT reconcil*) OR ("near" NEXT miss*)) OR [mh "patient harm"] OR [mh "patient safety"] OR [mh "Diagnostic Errors"] | | 53,707 |  |  |
| **6** | (efficien* OR economic* OR cost* OR expenditure* OR charge* OR (number NEAR/3 appointment*) OR (number NEAR/3 admission*) OR (number NEAR/3 consultation*)) | | 160,993 |  |  |
| **7** | ((wait* NEXT list*) OR (wait* NEXT time*) OR timeliness OR time?to?treatment) OR [mh "Waiting Lists"] | | 14,665 |  |  |
| **8** | ((health* OR "health care" OR access) NEAR/3 (equity OR disparit* OR inequit* OR inequalit* OR equality OR gap)) OR [mh "Health Equity"] | | 4,891 |  |  |
| **9** | 3 OR 4 OR 5 OR 6 OR 7 OR 8 | | 697,615 |  |  |
| **10** | 1 AND 2 AND 9 | | 268 |  |  |
| **11** | 10 with Publication Year from 2005 to 2025, in Trials | | 198 |  |  |

| **CINAHL** | |  |  |
| --- | --- | --- | --- |
| # | Query | Limiters/Expanders | Results |
| S1 | (MH "Telemedicine+" OR MH "Teleconsultation") |  | 29,887 |
| S2 | ((online OR phone* OR remote* OR tele* OR video* OR virtual*) N2 (appointment* OR care OR consult* OR hospital* OR monitor* OR visit* OR ward*)) |  | 23,218 |
| S3 | ("digital health" OR dhealth OR d-health OR "home monitor*" OR "mobile health" OR mHealth OR m-Health OR "long distance consultation*" OR teleconsult* OR tele-consult* OR telemonitor* OR tele-monitor* OR telehealth* OR "tele health*" OR tele-health* OR telemetry OR tele-homecare OR telehomecare OR "remote surveillance" OR "remotely delivered" OR "tele nursing" OR telenursing OR "self management" OR "self-management" OR "smartphone* app*" OR mobile*) |  | 116,711 |
| S4 | S1 OR S2 OR S3 |  | 141,497 |
| S5 | (MH "Diabetes Mellitus, Gestational+" OR MH "Diabetes Mellitus, In Pregnancy+") |  | 9,445 |
| S6 | (pregnan* N3 (gly* OR diabet* OR DM OR GDM)) |  | 7,210 |
| S7 | (gestational diabetes OR pregestational diabetes OR pre-gestational diabet* OR maternal diabet*) |  | 14,857 |
| S8 | S5 OR S6 OR S7 |  | 17,374 |
| S9 | (((experience* OR satisfaction) N4 (patient* OR consumer* OR client* OR survey* OR questionnaire*)) OR PREM* OR "patient-reported experience measure*" OR patient-cent#red* OR person-cent#red*) |  | 338,357 |
| S10 | ((health OR clinical* OR treatment*) N3 (outcome* OR effective* OR efficacy)) |  | 747,178 |
| S11 | (patient N3 (safety OR harm)) OR misdiagnos* OR "safety manag*" OR (accident* N2 prevent*) OR error* OR "diagnostic error*" OR "medication reconcil*" OR "near miss*") |  | 244,616 |
| S12 | (efficiency OR economic* OR cost* OR expenditure* OR charge* OR (number N3 appointment*) OR (number N3 admission*) OR (number N3 consultation*)) |  | 550,490 |
| S13 | ("wait* list*" OR "wait* time*" OR timeliness OR time-to-treatment OR "time to treatment") |  | 21,673 |
| S14 | ((health* OR "health care" OR access) N3 (equity OR disparit* OR inequit* OR inequalit* OR equality OR gap)) |  | 68,134 |
| S15 | S9 OR S10 OR S11 OR S12 OR S13 OR S14 |  | 1,719,237 |
| S16 | S4 AND S8 AND S15 |  | 156 |
| S17 | S4 AND S8 AND S15 | Limiters - Publication Date: 20050101-20251231 | 149 |

# Appendix B – Risk of Bias

**Figure B1:** Risk of bias using Risk of Bias 2 (RoB2) tool for randomised studies **(A)** Traffic light plot **(B)** Summary plot

**Figure B2:** Risk of bias using Risk Of Bias In Non-randomised Studies - of Interventions (ROBINS-I) tool for non-randomised studies **(A)** Traffic light plot **(B)** Summary plot

# Appendix C – Efficiency Results

**Table C1:** Summary of cost relating outcomes of remote versus usual care

| Author, year | Outcomes | Remote care | Usual care | Mean Difference  (95% CI), p-value |
| --- | --- | --- | --- | --- |
| Laurie et al., 2023 | Total average cost to service (AU$) | $129·39 | $143·92 | -$14·53, NR |
|  | Total average cost per patient (AU$) | $139·92 | $706·48 | - $566·56, NR |
| Lemelin et al., 2020 | Specific cost for GDM medical care pp (CAN$) | $170·89 | $307·89 | -$137·00, ***p* < 0·001** |
|  | Cost for nursing care for GDM pp (CAN$) | $256·51 | $143·11 | +113·40, ***p* < 0·001** |
|  | Cost of nutritional intervention pp (CAN$) | $28·89 | $76·39 | - $47·50, ***p* < 0·001** |
|  | Administrative cost pp (CAN$) | $212·11 | $273·47 | -$61·36, ***p* < 0·001** |
|  | Total cost for GDM management pp (CAN$) | $875·38 | $1,043·13 | -$167·75, *p* = 0·003 |
| Mackillop et al., 2018 | Total mean care cost per delivery | £5,697 ± 3,068 | £6,741 ± 464 | -£1,044 (-£2,186 to £99) |
| Rasekaba et al., 2018 | Total cost of unplanned appointments (AU$) | $968·5 ± 1,048·4 | $675·3 ± 972·7 | -$293, *p* = 0·05 |
|  | Total cost of planned appointments (AU$) | $1,735 ± 1,554·6 | $1,869 ± 1,175·3 | -$134, *p* = 0·23 |
| Sung et al., 2021 | Medication cost pp (US$) | $637 | US$1,104 | -$467 (-$4,089 to -$1,507)  ***p* < 0·01** |
|  | Inpatient facilities pp (US$) | $3,384 | $5,159 | -$1,775 (-$2,747 to -$803)  ***p* < 0·01** |
|  | Outpatient facilities pp (US$) | $694 | $1,026 | -$332 (-$492 to $173)  ***p* < 0·01** |
|  | Medical Professional pp (US$) | $3,080 | $3,291 | -$211 (-$832 to -$104), NS |
|  | Dental cost pp (US$) | $51 | $63 | -$12 (-$51 to $29), NS |
|  | Overall maternal care expenditure pp (US$) | $7,846 | $10,644 | -$2,798, ***p* < 0·01** |
| Syrop et al., 2020 | Mean maternal facilities cost pp (US$) | $9,512 | $7,828 | $1,684, *p* = 0·13 |
| Abbreviations: CI = Confidence Interval; GDM = Gestational Diabetes Mellitus; pp = per patient | | | | |

**Table C2:** Summary of appointment frequency and attendance relating outcomes of remote versus usual care

| Author, year | Outcomes | Remote care | Usual care | p-value |  |
| --- | --- | --- | --- | --- | --- |
| Amylidi-Mohr et al., 2025 | Appointments | **8·51 ± 2·8** | **8·04 ± 2·6** | **NS** |  |
| Berezowsky et al., 2024 | Appointments | **15·4 ± 6·2** | **14·6 ± 6·5** | *p =* 0·11 |  |
|  | Ultrasound visits | 5·9 ± 2·5 | 6·4 ± 3·2 | ***p =* 0·03** |  |
| Carral et al., 2023 | GDM unit visits | **3·2 ± 2·3** | **5·9 ± 3·2** | ***p* < 0·001** |  |
| Chatzakis et al., 2022 | Clinic visits | **2·9 ± 0·7** | **4·1 ± 1·1** | ***p* < 0·05** |  |
| Coveney et al., 2026 | DNA rates | 10·4% | 9·5% | NR |  |
| Clark et al., 2023 | Missed appointments | 1·21% | 6·15% | ***p* < 0·001** |  |
| Dolatabi et al., 2025 | Endocrinologist led appointment | 45·5% | 14·4% | NR |  |
| Fincher et al., 2025 | Pregnancy visits | 12·6 ± 5·35 | 10·8 ± 5·01 | *p* = 0·232 |  |
| Given et al., 2015 | Appointment attendance | 97·8% ± 6·1 | 92·6% ± 18·2 | NR |  |
| Guo et al., 2019 | OP visits | **8·1 ± 1·3** | **11·2 ± 1·1** | ***p* < 0·001** |  |
| Hyams et al., 2024 | Number of interactions | 7 (5–10) | 5 (3–6) | ***p* < 0·001** |  |
| Kantorowska et al., 2023 | Number of interactions | 12 (9-17) | 8 (5-12) | ***p* < 0·001** |  |
| Lemelin et al., 2020 | Medical visits | **1·5 ± 1·0** | **3·3 ± 1·1** | ***p* < 0·001** |  |
| Mackillop et al., 2018 | Hospital doctor visits | **4·65 ± 2·89** | **5·06 ± 2·86** | NS |  |
| Montori et al., 2023 | Total consultation time (mins) | **65 ± 21·9** | **97 ± 0·8** | ***p* < 0·001** |  |
| Munda et al., 2023 | Number of visits | **7 [5–10]** | **4 [3–6·5]** | ***p* < 0·001** |  |
| Pérez-Ferre et al., 2010 | Number of contacts (insulin-treated women) | **15·05** | **9·11** | ***p* < 0·001** |  |
|  | Total contact time per insulin-treated woman (hours) | **3·8** | **4·6** | ***p* < 0·001** |  |
| Rasekaba et al., 2018 | Number of appointments | **8 ± 7** | **8 ± 6** | *p* = 0·843 |  |
|  | Planned appointments | **4 ± 5** | **8 ± 10** | *p* = 0·096 |  |
|  | Unplanned appointments | 2 ± 4 | 1 ± 3 | *p* = 0·130 |  |
| Shashikumar et al., 202 | Scheduled appointments | 5 (4–8) | 4 (3–7) | ***p* < 0·0001** |  |
| Sung et al., 2021 | Prenatal visits | **10·45** | **11·31** | NS |  |
| Abbreviations: GDM = Gestational Diabetes Mellitus; NR = Not Reported; NS = Not Significant; OP = Outpatient | | | | | |

# Appendix D – Sensitivity Analysis By Diabetes Type

The tables below outline the sensitivity analysis for the effectiveness and safety outcomes by diabetes type· Table D1-D4 represent the meta-analysis when restricting to GDM relating studies only. There was only one study that separated T1DM (Dalfrà et al., 2009), thus not sensitivity analysis was undertaken for T1DM. Four studies reported jointly GDM, T1DM, T2DM thus no sensitivity analyses could be undertaken for those (Bartholomew et al. 2015, Kantorowska et al. 2023, Shashikumar et al. 2022, Syrop et al. 2020). Lastly, three studies (Carral et al. 2023, Fincher et al. 2025, Sung et al. 2021) reported combined T1DM and T2DM, which would not be appropriate to meta-analyse.

**Table D1:** Summary of meta-analysis results for maternal outcomes relating to clinical effectiveness comparing remote care to usual care for GDM only

| **Outcome** | **Study Design** | ***k*** | **Observations** | **Pooled effect** | | | **Heterogeneity I^2^** |
| --- | --- | --- | --- | --- | --- | --- | --- |
|  |  |  |  | **Effect Size** | **95% CI** | **p-value** |  |
| Elective c-section | RCT | 6 | 1,144 | RR = 1·00 | 0·75, 1·34 | 0·9901 | 16·7% |
|  | Non-RCT | 4 | 6,250 | RR = 0·96 | 0·73, 1·27 | 0·7925 | 58·2% |
| C-section (not specified) | RCT | 10 | 1,299 | RR = 1·01 | 0·83, 1·22 | 0·9350 | 50·8% |
|  | Non-RCT | 12 | 14,134 | RR = 0·92 | 0·84, 1·01 | 0·0899 | 30·3% |
| Vaginal spontaneous | RCT | 8 | 1,690 | RR = 1·06 | 0·97, 1·415 | 0·1933 | 24·4% |
|  | Non-RCT | 8 | 17,695 | RR = 1·00 | 0·94, 1·06 | 0·9412 | 64·0% |
| Vaginal operative | RCT | 6 | 1,324 | RR = 1·05 | 0·64, 1·72 | 0·8603 | 36·3% |
|  | Non-RCT | 6 | 7,213 | RR = 1·08 | 0·78, 1·49 | 0·6549 | 50·5% |
| Gestational weight gain (kg) | RCT | 11 | 1,612 | MD = -0·67 | -1·35, 0·02 | 0·0559 | 91·4% |
|  | Non-RCT | 5 | 718 | MD = -1·06 | -2·26, 0·15 | 0·0865 | 51·0% |
| Insulin started | RCT | 10 | 1,462 | RR = 1·07 | 0·74, 1·54 | 0·7168 | 59·8% |
|  | Non-RCT | 15 | 20,038 | RR = 1·02 | 0·90, 1·15 | 0·7742 | 72·2% |
| Metformin started | RCT | 2 | 561 | RR = 0·66 | 0·32, 1·37 | 0·2648 | 0·0% |
|  | Non-RCT | 5 | 16,638 | RR = 1·05 | 0·80, 1·38 | 0·7396 | 95·0% |
| Pre-eclampsia / Eclampsia | RCT | 2 | 419 | RR = 1·34 | 0·52, 3·46 | 0·5415 | 0·0% |
|  | Non-RCT | 5 | 2,385 | RR = 1·45 | 0·82, 2·59 | 0·2031 | 0·0% |
| GHTN | RCT | 3 | 264 | RR = 0·93 | 0·15, 5·60 | 0·9357 | 0·0% |
|  | Non-RCT | 5 | 1,177 | RR = 0·94 | 0·55, 1·60 | 0·8106 | 0·0% |
| Preeclampsia/Eclampsia/ /GHTN | RCT | 9 | 1,387 | RR = 1·10 | 0·68, 1·77 | 0·7099 | 0·0% |
|  | Non-RCT | 8 | 7,699 | RR = 1·12 | 0·91, 1·37 | 0·3015 | 0·0% |
| HbA1c (%) post-partum | RCT | 9 | 1,151 | MD = -0·12 | -0·29, 0·04 | 0·1430 | 82·9% |
|  | Non-RCT | 5 | 593 | MD = -0·09 | -0·15, -0·02 | **0·0099** | 41·4% |
| Post-partum FPG (mmol/L) | RCT | 8 | 766 | MD = -0·53 | -1·44, 0·37 | 0·2467 | 99·4% |
|  | Non-RCT | 3 | 349 | MD = -0·34 | -0·86, 0·18 | 0·2046 | 95·0% |
| Post-partum 2-hr PPG (mmol/L) | RCT | 6 | 522 | MD = -0·27 | -0·60, 0·07 | 0·1182 | 64·1% |
|  | Non-RCT | 2 | 152 | MD = -1·04 | -1·59, -0·48 | **0·0002** | 85·3% |
| Abbreviations: C-section = Caesarean Section; FPG = Fasting Plasma Glucose; PPG = Post-Prandial Glucose; GHTN = Gestational Hypertension; RR = Relative Risk; MD = Mean Difference; CI = Confidence Interval | | | | | | | |

**Table D2:** Summary of meta-analysis results for neonatal outcomes relating to clinical effectiveness comparing remote care to usual care for GDM only

| **Outcome** | **Study Design** | ***k*** | **Observations** | **Pooled effect** | | | **Heterogeneity I^2^** |
| --- | --- | --- | --- | --- | --- | --- | --- |
|  |  |  |  | **Effect Size** | **95% CI** | **p-value** |  |
| Birthweight (grams) | RCT | 11 | 1,173 | MD = -0·95 | -78·04, 76·14 | 0·9807 | 57·4% |
|  | Non-RCT | 13 | 10,462 | MD = -15·89 | -9·08, 40·86 | 0·2123 | 13·9% |
| Macrosomia | RCT | 10 | 1,785 | RR = 0·77 | 0·55, 1·06 | 0·1121 | 0·0% |
|  | Non-RCT | 11 | 13,056 | RR = 0·95 | 0·74, 1·21 | 0·6776 | 0·0% |
| SGA | RCT | 2 | 308 | RR = 1·91 | 0·45, 8·03 | 0·3769 | 0·0% |
|  | Non-RCT | 3 | 15,182 | RR = 1·02 | 0·92, 1·12 | 0·7226 | 0·0% |
| LGA | RCT | 9 | 1,211 | RR = 0·99 | 0·77, 1·28 | 0·957 | 22·0% |
|  | Non-RCT | 8 | 18,412 | RR = 0·92 | 0·80, 1·07 | 0·2980 | 51·1% |
| GA at delivery | RCT | 11 | 1,329 | MD = 0·02 | -0·17, 0·21 | 0·8412 | 55·6% |
|  | Non-RCT | 10 | 9,208 | MD = 0·04 | -0·06, 0·14 | 0·4471 | 37·9% |
| PTB | RCT | 11 | 1,866 | RR = 0·97 | 0·67, 1·42 | 0·8922 | 2·2% |
|  | Non-RCT | 7 | 3,998 | RR = 0·88 | 0·69, 1·30 | 0·5209 | 25·5% |
| Abbreviations: C-section = Caesarean Section; FPG = Fasting Plasma Glucose; PPG = Post-Prandial Glucose; SGA = Small for Gestational Age; LGA = Large for Gestational Age; GA = Gestational Age; GHTN = Gestational Hypertension; PTB = Pre-term Birth; RR = Relative Risk; MD = Mean Difference; CI = Confidence Interval | | | | | | | |

**Table D3:** Summary of meta-analysis results for maternal outcomes relating to safety comparing remote care to usual care for GDM only

| **Outcome** | **Study Design** | ***k*** | **Observations** | **Pooled effect** | | | **Heterogeneity I^2^** |
| --- | --- | --- | --- | --- | --- | --- | --- |
|  |  |  |  | **Effect Size** | **95% CI** | **p-value** |  |
| Emergency c-section | RCT | 6 | 1,144 | RR = 0·59 | 0·38, 0·92 | **0·0211** | 47·1% |
|  | Non-RCT | 3 | 6,250 | RR = 0·94 | 0·71, 1·24 | 0·6531 | 37·2% |
| Induction of Labour | RCT | 4 | 805 | RR = 0·90 | 0·76, 1·06 | 0·1960 | 14·5% |
|  | Non-RCT | 7 | 9,196 | RR = 1·04 | 0·88, 1·23 | 0·6728 | 73·1% |
| PROM | RCT | 4 | 551 | RR = 1·34 | 0·45, 4·08 | 0·6081 | 10·4% |
|  | Non-RCT | 1 | 161 | RR = 0·76 | 0·34, 1·70 | 0·5039 | N/A |
| Polyhydramnios | RCT | 3 | 538 | RR = 1·08 | 0·74, 1·57 | 0·7006 | 21·6% |
|  | Non-RCT | 1 | 63 | RR = 1·03 | 0·07, 15·79 | 0·9818 | N/A |
| Abbreviations: C-section = Caesarean Section; PROM = Premature Rupture of Membranes; RR = Relative Risk; CI = Confidence Interval | | | | | | | |

**Table D4:** Summary of meta-analysis results for neonatal outcomes relating to safety comparing remote care to usual care for GDM only

| **Outcome** | **Study Design** | ***k*** | **Observations** | **Pooled effect** | | | **Heterogeneity I^2^** |
| --- | --- | --- | --- | --- | --- | --- | --- |
|  |  |  |  | **Effect Size** | **95% CI** | **p-value** |  |
| NICU | RCT | 10 | 1,647 | RR = 0·73 | 0·57, 0·92 | **0·0089** | 0·0% |
|  | Non-RCT | 6 | 17,358 | RR = 0·99 | 0·89, 1·09 | 0·7823 | 33·5% |
| Jaundice | RCT | 8 | 1,110 | RR = 0·97 | 0·72, 1·33 | 0·8676 | 0·0% |
|  | Non-RCT | 6 | 3,042 | RR = 1·11 | 0·48, 2·57 | 0·8137 | 76·2% |
| Hypoglycaemia | RCT | 12 | 1,736 | RR = 0·95 | 0·74, 1·24 | 0·7255 | 0·0% |
|  | Non-RCT | 7 | 8,072 | RR = 0·79 | 0·30, 2·05 | 0·6221 | 88·3% |
| Respiratory Distress | RCT | 7 | 1,159 | RR = 0·84 | 0·60, 1·17 | 0·2965 | 6·5% |
|  | Non-RCT | 5 | 6,410 | RR = 1·34 | 1·00, 1·78 | **0·0498** | 0·0% |
| Shoulder Dystocia | RCT | 4 | 1,038 | RR = 0·55 | 0·14, 2·14 | 0·3890 | 22·1% |
|  | Non-RCT | 7 | 8,925 | RR = 0·90 | 0·35, 2·33 | 0·8356 | 65·7% |
| Stillbirth | RCT | 0 | - |  |  |  |  |
|  | Non-RCT | 3 | 15,666 | RR = 0·74 | 0·38, 1·45 | 0·3874 | 0·0% |
| Abbreviations: NICU = Neonatal Intensive Care Unit; RR = Relative Risk; CI = Confidence Interval | | | | | | | |

# Appendix E – Sensitivity Analysis By Remote Care Type

The tables below outline the sensitivity analysis of effectiveness and safety outcomes by remote care type·

**Table E1:** Summary of meta-analysis results for maternal outcomes relating to clinical effectiveness comparing remote care to usual care by study design and remote care type

| **Outcome** | **Study Design x Remote Care Type** | ***k*** | **Observations** | **Pooled effect** | | | **Heterogeneity I^2^** |
| --- | --- | --- | --- | --- | --- | --- | --- |
|  |  |  |  | **Effect Size** | **95% CI** | **p-value** |  |
| Elective c-section | RCT - mHealth | 5 | 1,049 | RR = 0·95 | 0·69, 1·29 | 0·3185 | 15·0% |
|  | RCT - Web-based system | 1 | 95 | RR = 1·58 | 0·69, 3·62 | 0·2811 | N/A |
|  | Non-RCT – Hybrid Telemedicine | 1 | 775 | RR = 0·99 | 0·70, 1·40 | 0·9609 | N/A |
|  | Non-RCT – mHealth | 1 | 63 | RR = 0·88 | 0·49, 1·60 | 0·6853 | N/A |
|  | Non-RCT – Web-based System | 1 | 161 | RR = 0·58 | 0·34, 1·02 | 0·0578 | N/A |
|  | Non-RCT - Telemedicine | 2 | 5,617 | RR = 1·18 | 1·06, 1·30 | **0·0016** | 0·0% |
| C-section (not specified) | RCT – CGM | 2 | 405 | RR = 0·91 | 0·75, 1·10 | 0·3227 | 0·0% |
|  | RCT – Telemedicine | 1 | 47 | RR = 0·41 | 0·02, 9·58 | 0·5799 | N/A |
|  | RCT – mHealth | 5 | 714 | RR = 1·02 | 0·77, 1·38 | 0·8257 | 55·6% |
|  | RCT – Web-based System | 2 | 133 | RR = 1·08 | 0·46, 2·62 | 0·8633 | 81·8% |
|  | Non-RCT – Web-based system | 2 | 637 | RR = 0·92 | 0·77, 1·11 | 0·3983 | 0·0% |
|  | Non-RCT – Hybrid Telemedicine | 2 | 9,902 | RR = 0·96 | 0·91, 1·01 | 0·1263 | 0·0% |
|  | Non-RCT – Telemedicine | 2 | 847 | RR = 1·04 | 0·92, 1·18 | 0·5488 | 0·0% |
|  | Non-RCT – mHealth | 12 | 4,062 | RR = 0·87 | 0·77, 0·98 | **0·0209** | 17·8% |
| Vaginal spontaneous | RCT – CGM | 1 | 299 | RR = 1·17 | 0·93, 1·48 | 0·1838 | N/A |
|  | RCT – mHealth | 7 | 1,391 | RR = 1·04 | 0·95, 1·15 | 0·3653 | 28·8% |
|  | Non-RCT – Hybrid Telemedicine | 3 | 4,617 | RR = 1·05 | 1·01, 1·09 | **0·0131** | 0·0% |
|  | Non-RCT – mHealth | 6 | 2,068 | RR = 1·05 | 1·01, 1·10 | 0·4578 | 18·8% |
|  | Non-RCT – Web-based System | 1 | 533 | RR = 1·10 | 0·91, 1·32 | 0·3340 | N/A |
|  | Non-RCT - Telemedicine | 2 | 5,617 | RR = 0·95 | 0·84, 1·08 | 0·4385 | 36·2% |
| Vaginal operative | RCT – mHealth | 5 | 1,025 | RR = 1·32 | 0·87, 2·01 | 0·1903 | 0·0% |
|  | RCT – CGM | 1 | 299 | RR = 0·52 | 0·24, 1·13 | 0·0980 | N/A |
|  | Non-RCT – Hybrid Telemedicine | 1 | 775 | RR = 1·61 | 0·95, 2·74 | 0·0754 | N/A |
|  | Non-RCT – mHealth | 3 | 1,026 | RR = 1·06 | 0·72, 1·56 | 0·7565 | 0·0% |
|  | Non-RCT – Web-based system | 2 | 694 | RR = 0·90 | 0·31, 2·56 | 0·8376 | 59·7% |
|  | Non-RCT - Telemedicine | 2 | 5,617 | RR = 0·76 | 0·65, 0·90 | **0·0010** | 0·0% |
| Gestational weight gain (kg) | RCT – mHealth | 7 | 1,074 | MD = -0·79 | -1·66, 0·08 | 0·0734 | 94·7% |
|  | RCT – CGM | 2 | 405 | MD = -0·54 | -2·07, 0·98 | 0·4874 | 57·0% |
|  | RCT – Web-based System | 2 | 133 | MD = 0·53 | -2·27, 3·32 | 0·7078 | 0·0% |
|  | Non-RCT – Web-based System | 2 | 265 | MD = -0·17 | -1,54, 1·20 | 0·8087 | 0·0% |
|  | Non-RCT – mHealth | 5 | 458 | MD = -1·33 | -2·61, -0·06 | **0·0407** | 33·3% |
| Insulin started | RCT – CGM | 2 | 405 | RR = 1·50 | 0·70, 3·21 | 0·2918 | 71·4% |
|  | RCT – mHealth | 6 | 924 | RR = 0·81 | 0·53, 1·26 | 0·3529 | 51·9% |
|  | RCT – Web-based System | 2 | 133 | RR = 2·53 | 0·50, 12·68 | 0·2589 | 59·7% |
|  | Non-RCT – Web-based System | 3 | 771 | RR = 0·93 | 0·55, 1·58 | 0·7910 | 67·6% |
|  | Non-RCT – mHealth | 11 | 4,307 | RR = 1·03 | 0·95, 1·12 | 0·4593 | 1·5% |
|  | Non-RCT – Telemedicine | 3 | 6,098 | RR = 1·08 | 0·72, 1·61 | 0·7259 | 91·6% |
|  | Non-RCT – Hybrid Telemedicine | 1 | 9,771 | RR = 1·18 | 1·10, 1·16 | **<0·001** | N/A |
| Metformin started | RCT – mHealth | 2 | 561 | RR = 0·66 | 0·32, 1·37 | 0·2648 | 0·0% |
|  | Non-RCT – mHealth | 3 | 1,616 | RR = 1·08 | 0·88, 1·32 | 0·4603 | 39·4% |
|  | Non-RCT – Hybrid Telemedicine | 1 | 9,771 | RR = 1·35 | 1·16, 1·57 | <0·0001 | N/A |
|  | Non-RCT – Web-based system | 1 | 681 | RR = 1·00 | 0·75, 1·34 | 0·9938 | N/A |
|  | Non-RCT – Telemedicine | 1 | 5,251 | RR = 0·68 | 0·63, 0·74 | <0·0001 | N/A |
| Pre-eclampsia / Eclampsia | RCT – CGM | 1 | 299 | RR = 1·28 | 0·42, 3·95 | 0·6638 | N/A |
|  | RCT – mHealth | 1 | 120 | RR = 1·50 | 0·26, 8·67 | 0·6503 | N/A |
|  | Non-RCT – mHealth | 5 | 2,224 | RR = 1·45 | 0·82, 2·59 | 0·2031 | 0·0% |
|  | Non-RCT – Web-based System | 1 | 694 | RR = 0·39 | 0·22, 0·67 | **0·0007** | N/A |
| GHTN | RCT – Telemedicine | 1 | 47 | RR = 0·41 | 0·02, 9·58 | 0·5799 | N/A |
|  | RCT - mHealth | 2 | 217 | RR = 1·34 | 0·10, 18·69 | 0·2290 | 30·9% |
|  | Non-RCT – Web-based System | 3 | 798 | RR = 1·52 | 0·69, 3·35 | 0·3013 | 0·0% |
|  | Non-RCT – mHealth | 4 | 1,016 | RR = 0·97 | 0·56, 1·68 | 0·9078 | 0·0% |
|  | Non-RCT - Telemedicine | 1 | 366 | RR = 0·52 | 0·16, 1·70 | 0·2818 | N/A |
| Preeclampsia/Eclampsia/GHTN | RCT – CGM | 1 | 299 | RR = 1·28 | 0·42, 3·95 | 0·6638 | N/A |
|  | RCT – Telemedicine | 1 | 47 | RR = 0·41 | 0·02, 9·58 | 0·5799 | N/A |
|  | RCT – Web-based System | 2 | 133 | RR = 1·41 | 0·61, 3·23 | 0·4216 | 0·0% |
|  | RCT – mHealth | 5 | 908 | RR = 0·88 | 0·35, 2·17 | 0·7767 | 36·4% |
|  | Non-RCT – Web-based System | 3 | 798 | RR = 0·66 | 0·44, 1·00 | **0·0496** | 0·0% |
|  | Non-RCT – mHealth | 6 | 2,287 | RR = 1·19 | 0·89, 1·77 | 0·3945 | 0·0% |
|  | Non-RCT – Telemedicine | 2 | 5,617 | RR = 0·96 | 0·54, 1·69 | 0·6046 | 31·3% |
| HbA1c (%) post-partum | RCT – mHealth | 5 | 646 | MD = -0·18 | -0·43, 0·06 | 0·1364 | 89·1% |
|  | RCT – CGM | 2 | 405 | MD = -0·01 | -0·16, 0·13 | 0·8593 | 69·5% |
|  | RCT – Telemedicine | 1 | 47 | MD = 0·02 | -1·38, 1·42 | 0·9776 | N/A |
|  | RCT – Web-based System | 1 | 53 | MD = -0·10 | -1·04, 0·84 | 0·8354 | N/A |
|  | Non-RCT – Web-based System | 4 | 369 | MD = -0·07 | -0·15, 0·02 | 0·1400 | 0·0% |
|  | Non-RCT – mHealth | 4 | 387 | MD = -0·04 | -0·25, 0·17 | 0·7047 | 50·8% |
| Post-partum FPG (mmol/L) | RCT - mHealth | 6 | 633 | MD = -0·71 | -1·90, 0·48 | 0·2419 | 99·6% |
|  | RCT – Web-based System | 2 | 133 | MD = -0·02 | -0·22, 0·18 | 0·8449 | 0·0% |
|  | Non-RCT – mHealth | 3 | 349 | MD = -0·34 | -0·86, 0·18 | 0·2046 | 95·0% |
| Post-partum 2-hr PPG (mmol/L) | RCT - mHealth | 4 | 389 | MD = -0·52 | -1·14, 0·09 | 0·0966 | 63·6% |
|  | RCT – Web-based System | 2 | 133 | MD = -0·06 | -0·25, 0·14 | 0·5713 | 0·0% |
|  | Non-RCT – mHealth | 2 | 152 | MD = -1·04 | -1·59, -0·48 | **0·0002** | 85·3% |
| Abbreviations: C-section = Caesarean Section; FPG = Fasting Plasma Glucose; PPG = Post-Prandial Glucose; GHTN = Gestational Hypertension; RR = Relative Risk; MD = Mean Difference; CI = Confidence Interval | | | | | | | |

**Table E2:** Summary of meta-analysis results for neonatal outcomes relating to clinical effectiveness comparing remote care to usual care by study design and remote care type

| **Outcome** | **Study Design x Remote Care Type** | ***k*** | **Observations** | **Pooled effect** | | | **Heterogeneity I^2^** |
| --- | --- | --- | --- | --- | --- | --- | --- |
|  |  |  |  | **Effect Size** | **95% CI** | **p-value** |  |
| Birthweight (grams) | RCT – Telemedicine | 1 | 47 | MD = 285·00 | -22·62, 592·62 | 0·0694 | N/A |
|  | RCT – Web-based System | 3 | 228 | MD = 101·01 | -24·42, 226·44 | 0·1145 | 0·0% |
|  | RCT – mHealth | 6 | 792 | MD = -40·80 | -120·95, 39·35 | 0·3185 | 44·2% |
|  | RCT – CGM | 1 | 106 | MD = -175·21 | -372·19, 21·77 | 0·0813 | N/A |
|  | Non-RCT – Hybrid Telemedicine | 1 | 775 | MD = -9·00 | -85·59, 67·59 | 0·8179 | N/A |
|  | Non-RCT – mHealth | 10 | 4,275 | MD = -31·94 | -66·44, 2·56 | 0·0630 | 20·2% |
|  | Non-RCT – Web-based system | 1 | 161 | MD = -67·18 | -135·03, 0·66 | 0·6136 | N/A |
|  | Non-RCT - Telemedicine | 1 | 5,251 | MD = 7·00 | -23·38, 37·38 | 0·6516 | N/A |
| Macrosomia | RCT – CGM | 2 | 405 | RR = 0·79 | 0·39, 1·61 | 0·5186 | 0·0% |
|  | RCT – mHealth | 6 | 1,238 | RR = 0·67 | 0·46, 0·98 | **0·0411** | 0·0% |
|  | RCT – Telemedicine | 1 | 47 | RR = 3·71 | 0·83, 16·54 | 0·0850 | N/A |
|  | RCT – Web-based System | 1 | 95 | RR = 1·67 | 0·18, 15·46 | 0·6505 | N/A |
|  | Non-RCT – mHealth | 8 | 2,522 | RR = 0·91 | 0·69, 1·22 | 0·5355 | 0·0% |
|  | Non-RCT – Hybrid Telemedicine | 2 | 9,901 | RR = 0·94 | 0·56, 1·58 | 0·8076 | 0·0% |
|  | Non-RCT – Telemedicine | 1 | 503 | RR = 1·78 | 0·71, 4·42 | 0·2188 | N/A |
|  | Non-RCT – Web-based System | 1 | 161 | RR = 0·51 | 0·04, 5·47 | 0·5752 | N/A |
| SGA | RCT – CGM | 1 | 106 | RR = 1·08 | 0·16, 7·37 | 0·9386 | N/A |
|  | RCT – mHealth | 1 | 202 | RR = 3·92 | 0·45, 33·95 | 0·2142 | N/A |
|  | Non-RCT – Web-based System | 2 | 265 | RR = 1·30 | 0·55, 3·09 | 0·5546 | 0·0% |
|  | Non-RCT – Hybrid Telemedicine | 1 | 9,771 | RR = 1·04 | 0·93, 1·16 | 0·5007 | N/A |
|  | Non-RCT – Telemedicine | 1 | 5,251 | RR = 0·92 | 0·73, 1·17 | 0·4909 | N/A |
|  | Non-RCT – mHealth | 1 | 251 | RR = 0·74 | 0·28, 1·91 | 0·5290 | N/A |
| LGA | RCT – CGM | 2 | 405 | RR = 0·77 | 0·53, 1·11 | 0·1636 | 13·9% |
|  | RCT – Web-based System | 2 | 133 | RR = 1·58 | 0·78, 3·22 | 0·2052 | 0·0% |
|  | RCT – mHealth | 5 | 673 | RR = 1·15 | 0·77, 1·72 | 0·4894 | 10·8% |
|  | Non-RCT – Web-based System | 3 | 798 | RR = 0·93 | 0·61, 1·43 | 0·7553 | 0·0% |
|  | Non-RCT – Hybrid Telemedicine | 2 | 10,546 | RR = 0·82 | 0·73, 0·93 | **0·0013** | 0·0% |
|  | Non-RCT – Telemedicine | 3 | 5,961 | RR = 1·04 | 0·95, 1·14 | 0·3630 | 56·1% |
|  | Non-RCT – mHealth | 5 | 2,714 | RR = 0·93 | 0·75, 1·14 | 0·4895 | 14·6% |
| GA at delivery | RCT – CGM | 2 | 405 | MD = -0·15 | -0·39, 0·09 | 0·2229 | 0·0% |
|  | RCT – Telemedicine | 1 | 47 | MD = 0·10 | -0·51, 0·71 | 0·7462 | N/A |
|  | RCT – mHealth | 5 | 649 | MD = 0·04 | -0·28, 0·36 | 0·8109 | 65·7% |
|  | RCT – Web-based System | 3 | 228 | MD = 0·13 | -0·42, 0·68 | 0·6416 | 57·1% |
|  | Non-RCT – Hybrid Telemedicine | 1 | 775 | MD = 0·00 | -0·20, 0·20 | 1·0000 | N/A |
|  | Non-RCT – Web-based System | 3 | 798 | MD = -0·04 | -0·48, 0·40 | 0·8530 | 72·4% |
|  | Non-RCT – mHealth | 10 | 3,354 | MD = 0·07 | -0·02, 0·15 | 0·1338 | 0·0% |
|  | Non-RCT - Telemedicine | 2 | 5,617 | MD = -0·07 | -0·21, 0·08 | 0·0506 | 24·1% |
| PTB | RCT – CGM | 1 | 405 | RR = 1·83 | 0·34, 9·86 | 0·4800 | N/A |
|  | RCT – Telemedicine | 1 | 47 | RR = 0·25 | 0·01, 4·87 | 0·3575 | N/A |
|  | RCT – Web-based System | 2 | 133 | RR = 0·72 | 0·32, 1·63 | 0·4342 | 0·0% |
|  | RCT - mHealth | 7 | 1,281 | RR = 1·03 | 0·69, 1·54 | 0,8685 | 20·3% |
|  | Non-RCT – Hybrid Telemedicine | 1 | 775 | RR = 0·82 | 0·46, 1·43 | 0·4780 | N/A |
|  | Non-RCT – Web-based System | 2 | 265 | RR = 1·68 | 0·29, 9·71 | 0·5645 | 31·1% |
|  | Non-RCT – mHealth | 6 | 3,313 | RR = 0·81 | 0·56, 1·17 | 0·0513 | 20·4% |
|  | Non-RCT - Telemedicine | 2 | 710 | RR = 1·04 | 0·73, 1·48 | 0·8266 | 0·0% |
| Abbreviations: C-section = Caesarean Section; FPG = Fasting Plasma Glucose; PPG = Post-Prandial Glucose; SGA = Small for Gestational Age; LGA = Large for Gestational Age; GA = Gestational Age; GHTN = Gestational Hypertension; PTB = Pre-term Birth; RR = Relative Risk; MD = Mean Difference; CI = Confidence Interval | | | | | | | |

**Table E3:** Summary of meta-analysis results for maternal outcomes relating to safety comparing remote care to usual care by study design and remote care type

| **Outcome** | **Study Design x Remote Care Type** | ***k*** | **Observations** | **Pooled effect** | | | **Heterogeneity I^2^** |
| --- | --- | --- | --- | --- | --- | --- | --- |
|  |  |  |  | **Effect Size** | **95% CI** | **p-value** |  |
| Emergency c-section | RCT - mHealth | 5 | 1,049 | RR = 0·53 | 0·34, 0·85 | **0·0077** | 45·7% |
|  | RCT – Web-based system | 1 | 95 | RR = 1·23 | 0·46, 3·24 | 0·6804 | N/A |
|  | Non-RCT – Hybrid Telemedicine | 1 | 775 | RR = 0·79 | 0·50, 1·23 | 0·2934 | N/A |
|  | Non-RCT – Telemedicine | 1 | 5,251 | RR = 0·65 | 0·30, 1·42 | 0·1886 | N/A |
|  | Non-RCT Web-based System | 1 | 161 | RR = 1·07 | 0·97, 1·19 | 0·2795 | N/A |
| Induction of Labour | RCT – mHealth | 3 | 506 | RR = 0·96 | 0·73, 1·26 | 0·7963 | 34·0% |
|  | RCT – CGM | 1 | 299 | RR = 0·83 | 0·64, 1·09 | 0·1814 | N/A |
|  | Non-RCT – mHealth | 5 | 3,170 | RR = 1·00 | 0·78, 1·27 | 0·9894 | 72·3% |
|  | Non-RCT – Hybrid Telemedicine | 1 | 775 | RR = 1·27 | 1·06, 1·51 | **0·0085** | N/A |
|  | Non-RCT – Telemedicine | 1 | 5,251 | RR = 1·00 | 0·94, 1·06 | 0·9549 | N/A |
|  | Non-RCT – Web-based System | 1 | 533 | RR = 1·13 | 0·88, 1·44 | 0·3457 | N/A |
| PROM | RCT – CGM | 1 | 299 | RR = 2·75 | 0·29, 26·14 | 0·3786 | N/A |
|  | RCT – Web-based System | 2 | 133 | RR = 0·94 | 0·05, 16·95 | 0·9668 | 59·2% |
|  | RCT – mHealth | 1 | 119 | RR = 1·58 | 0·27, 9·10 | 0·6102 | N/A |
|  | Non-RCT – Web-based System | 1 | 161 | RR = 0·76 | 0·34, 1·70 | 0·5039 | N/A |
| Polyhydramnios | RCT – CGM | 1 | 299 | RR = 1·10 | 0·75, 1·62 | 0·6293 | N/A |
|  | RCT – mHealth | 2 | 239 | RR = 0·54 | 0·04, 6·96 | 0·6397 | 57·5% |
|  | Non-RCT – mHealth | 1 | 63 | RR = 1·03 | 0·07, 15·79 | 0·9818 | N/A |
| Abbreviations: C-section = Caesarean Section; PROM = Premature Rupture of Membranes; RR = Relative Risk; CI = Confidence Interval | | | | | | | |

**Table E4:** Summary of meta-analysis results for neonatal outcomes relating to safety comparing remote care to usual care by study design and remote care type

| **Outcome** | **Study Design x Remote Care Type** | ***k*** | **Observations** | **Pooled effect** | | | **Heterogeneity I^2^** |
| --- | --- | --- | --- | --- | --- | --- | --- |
|  |  |  |  | **Effect Size** | **95% CI** | **p-value** |  |
| NICU | RCT – CGM | 1 | 299 | RR = 1·15 | 0·32, 4·18 | 0·8368 | N/A |
|  | RCT – mHealth | 6 | 1,168 | RR = 0·64 | 0·49, 0·85 | **0·0016** | 0·0% |
|  | RCT – Telemedicine | 1 | 47 | RR = 1·24 | 0·60, 2·55 | 0·5627 | N/A |
|  | RCT – Web-based System | 2 | 133 | RR = 0·89 | 0·39, 2·05 | 0·7758 | 9·6% |
|  | Non-RCT – Hybrid Telemedicine | 2 | 10,546 | RR = 1·01 | 0·85, 1·41 | 0·5616 | 18·4% |
|  | Non-RCT – mHealth | 4 | 1,359 | RR = 0·95 | 0·65, 1·41 | 0·8145 | 62·0% |
|  | Non-RCT – Telemedicine | 3 | 6,120 | RR = 0·91 | 0·78, 1·06 | 0·2311 | 0·0% |
|  | Non-RCT – Web-based System | 1 | 533 | RR = 0·81 | 0·57, 1·16 | 0·2496 | N/A |
| Jaundice | RCT – Telemedicine | 1 | 47 | RR = 1·55 | 0·75, 3·21 | 0·2413 | N/A |
|  | RCT – Web-based System | 2 | 133 | RR = 0·55 | 0·17, 1·84 | 0·3359 | 0·0% |
|  | RCT – mHealth | 5 | 930 | RR = 0·92 | 0·64, 1·31 | 0·6342 | 0·0% |
|  | Non-RCT – Hybrid Telemedicine | 2 | 906 | RR = 0·59 | 0·30, 1·15 | 0·1180 | 0·0% |
|  | Non-RCT – Web-based System | 1 | 161 | RR = 0·67 | 0·46, 0·99 | 0·3671 | N/A |
|  | Non-RCT – mHealth | 3 | 1,975 | RR = 1·77 | 0·60, 5·23 | 0·6164 | 2·8% |
| Hypoglycaemia | RCT – CGM | 2 | 405 | RR = 0·79 | 0·39, 1·61 | 0·5164 | 0·0% |
|  | RCT – Telemedicine | 1 | 47 | RR = 0·83 | 0·27, 2·55 | 0·7386 | N/A |
|  | RCT – Web-based System | 2 | 133 | RR = 1·19 | 0·43, 3·28 | 0·7329 | 0·0% |
|  | RCT – mHealth | 7 | 1,151 | RR = 0·98 | 0·72, 1·34 | 0·8592 | 5·4% |
|  | Non-RCT – Hybrid Telemedicine | 2 | 906 | RR = 1·57 | 0·12, 21·10 | 0·7407 | 81·1% |
|  | Non-RCT – Web-based System | 3 | 798 | RR = 0·78 | 0·41, 1·50 | 0·4357 | 58·5% |
|  | Non-RCT – mHealth | 4 | 1,804 | RR = 0·35 | 0·11, 1·04 | 0·0883 | 78·8% |
|  | Non-RCT - Telemedicine | 2 | 5,617 | RR = 0·88 | 0·70, 1·10 | 0·2382 | 0·0% |
| Respiratory Distress | RCT – CGM | 1 | 299 | RR = 2·75 | 0·76, 9·96 | 0·1234 | N/A |
|  | RCT – mHealth | 4 | 727 | RR = 0·78 | 0·53, 1·13 | 0·1862 | 0·0% |
|  | RCT – Web-based System | 2 | 133 | RR = 0·70 | 0·27, 1·81 | 0·4581 | 0·0% |
|  | Non-RCT – Hybrid Telemedicine | 1 | 775 | RR = 1·65 | 0·78, 3·50 | 0·1867 | N/A |
|  | Non-RCT – Web-based System | 2 | 694 | RR = 0·73 | 0·41, 1·32 | 0·3021 | 0·0% |
|  | Non-RCT – mHealth | 3 | 273 | RR = 1·51 | 0·62, 3·71 | 0·3676 | 18·6% |
|  | Non-RCT - Telemedicine | 1 | 5,251 | RR = 1·30 | 0·94, 1·79 | 0·1126 | N/A |
| Shoulder Dystocia | RCT – CGM | 1 | 299 | RR = 0·92 | 0·19, 4·47 | 0·9143 | N/A |
|  | RCT – Telemedicine | 1 | 47 | RR = 0·07 | 0·00, 1·05 | 0·0543 | N/A |
|  | RCT – mHealth | 2 | 692 | RR = 0·99 | 0·10, 9·40 | 0·9932 | 0·0% |
|  | Non-RCT – Hybrid Telemedicine | 1 | 775 | RR = 5·03 | 1·67, 15·19 | **0·0042** | N/A |
|  | Non-RCT – mHealth | 4 | 2,285 | RR = 0·53 | 0·24, 1·13 | 0·1006 | 0·0% |
|  | Non-RCT – Telemedicine | 2 | 5,754 | RR = 0·40 | 0·05, 3·01 | 0·3710 | 74·1% |
|  | Non-RCT – Web-based System | 2 | 694 | RR = 1·16 | 0·12, 10·86 | 0·8949 | 39·3% |
| Stillbirth | RCT | 0 | - |  |  |  |  |
|  | Non-RCT – Hybrid Telemedicine | 1 | 9,771 | RR = 0·99 | 0·39, 2·51 | 0·9851 | N/A |
|  | Non-RCT – Telemedicine | 1 | 5,251 | RR = 0·61 | 0·27, 1·40 | 0·3024 | N/A |
|  | Non-RCT – mHealth | 1 | 644 | RR = 0·42 | 0·04, 4·64 | 0·4810 | N/A |
| Abbreviations: NICU = Neonatal Intensive Care Unit; RR = Relative Risk; CI = Confidence Interval | | | | | | | |

# Appendix F – Sensitivity Analysis By Risk of Bias

The tables below outline the sensitivity analysis of effectiveness and safety outcomes by excluding high risk studies·

**Table F1:** Summary of meta-analysis results for maternal outcomes relating to clinical effectiveness comparing remote care to usual care for non-high risk studies

| **Outcome** | **Study Design** | ***k*** | **Observations** | **Pooled effect** | | | **Heterogeneity I^2^** |
| --- | --- | --- | --- | --- | --- | --- | --- |
|  |  |  |  | **Effect Size** | **95% CI** | **p-value** |  |
| Elective c-section | RCT | 3 | 418 | RR = 0·90 | 0·56, 1·44 | 0·6632 | 41·2% |
|  | Non-RCT | 4 | 6,455 | RR = 1·15 | 1·05, 1·27 | **0·0036** | 0·0% |
| C-section (not specified) | RCT | 7 | 1,042 | RR = 1·03 | 0·86, 1·24 | 0·7581 | 46·9% |
|  | Non-RCT | 13 | 14,768 | RR = 0·96 | 0·90, 1·02 | **0·1733** | 23·8% |
| Vaginal spontaneous | RCT | 5 | 988 | RR = 1·06 | 0·90, 1·25 | 0·4997 | 52·7% |
|  | Non-RCT | 9 | 18,542 | RR = 1·01 | 0·95, 1·06 | 0·8598 | 54·3% |
| Vaginal operative | RCT | 3 | 622 | RR = 1·21 | 0·43, 3·45 | 0·7192 | 70·7% |
|  | Non-RCT | 5 | 7,632 | RR = 0·88 | 0·61, 1·26 | 0·4923 | 59·3% |
| Gestational weight gain (kg) | RCT | 5 | 810 | MD = -0·23 | -0·98, 0·52 | 0·5514 | 51·9% |
|  | Non-RCT | 5 | 633 | MD = -1·27 | -2·53, -0·01 | **0·0480** | 35·9% |
| Insulin started | RCT | 5 | 727 | RR = 1·05 | 0·57, 1·93 | 0·8853 | 72·5% |
|  | Non-RCT | 14 | 20,407 | RR = 1·04 | 0·93, 1·16 | 0·4740 | 75·5% |
| Metformin started | RCT | 0 | - |  |  |  |  |
|  | Non-RCT | 5 | 17,073 | RR = 1·01 | 0·79, 1·29 | 0·9631 | 94·9% |
| Pre-eclampsia / Eclampsia | RCT | 2 | 419 | RR = 1·34 | 0·52, 3·46 | 0·5415 | 0·0% |
|  | Non-RCT | 4 | 2,645 | RR = 1·04 | 0·40, 2·69 | 0·9328 | 76·0% |
| GHTN | RCT | 3 | 264 | RR = 0·93 | 0·15, 5·60 | 0·9357 | 0·0% |
|  | Non-RCT | 5 | 1,907 | RR = 1·09 | 0·70, 1·70 | 0·7067 | 0·0% |
| Preeclampsia/Eclampsia/ /GHTN | RCT | 5 | 766 | RR = 0·96 | 0·43, 2·10 | 0·9145 | 0·0% |
|  | Non-RCT | 8 | 8,429 | RR = 0·99 | 0·74, 1·32 | 0·9451 | 16·6% |
| HbA1c (%) post-partum | RCT | 5 | 677 | MD = -0·10 | -0·35, 0·15 | 0·4332 | 90·6% |
|  | Non-RCT | 5 | 439 | MD = -0·02 | -0·19, 0·16 | 0·8629 | 47·7% |
| Post-partum FPG (mmol/L) | RCT | 3 | 428 | MD = -1·21 | -3·66, 1·24 | 0·3317 | 99·8% |
|  | Non-RCT | 2 | 297 | MD = -0·34 | -1·21, 0·53 | 0·4475 | 97·4% |
| Post-partum 2-hr PPG (mmol/L) | RCT | 2 | 308 | MD = -0·29 | -0·43, -0·16 | **<0·0001** | 0·0% |
|  | Non-RCT | 1 | 100 | MD = -1·36 | -1·79, -0·94 | **<0·0001** | N/A |
| Abbreviations: C-section = Caesarean Section; FPG = Fasting Plasma Glucose; PPG = Post-Prandial Glucose; GHTN = Gestational Hypertension; RR = Relative Risk; MD = Mean Difference; CI = Confidence Interval | | | | | | | |

**Table F2:** Summary of meta-analysis results for neonatal outcomes relating to clinical effectiveness comparing remote care to usual care for non-high risk studies

| **Outcome** | **Study Design** | ***k*** | **Observations** | **Pooled effect** | | | **Heterogeneity I^2^** |
| --- | --- | --- | --- | --- | --- | --- | --- |
|  |  |  |  | **Effect Size** | **95% CI** | **p-value** |  |
| Birthweight (grams) | RCT | 8 | 892 | MD = -2·50 | -81·55, 76·55 | 0·9507 | 57·7% |
|  | Non-RCT | 13 | 10,462 | MD = -20·22 | -46·18, 5·75 | 0·2882 | 15·0% |
| Macrosomia | RCT | 6 | 935 | RR = 0·91 | 0·60, 1·38 | 0·6567 | 0·0% |
|  | Non-RCT | 9 | 12,815 | RR = 0·97 | 0·75, 1·24 | 0·7778 | 0·0% |
| SGA | RCT | 2 | 308 | RR = 1·91 | 0·45, 8·03 | 0·3769 | 0·0% |
|  | Non-RCT | 3 | 15,125 | RR = 1·02 | 0·92, 1·12 | 0·7158 | 0·0% |
| LGA | RCT | 6 | 930 | RR = 0·98 | 0·70, 1·18 | 0·9039 | 0·0% |
|  | Non-RCT | 10 | 19,547 | RR = 0·94 | 0·82, 1·07 | 0·3584 | 52·3% |
| GA at delivery | RCT | 8 | 1,072 | MD = -0·04 | -0·25, 0·17 | 0·6935 | 63·7% |
|  | Non-RCT | 12 | 10,022 | MD = -0·01 | -0·09, 0·08 | 0·8980 | 26·8% |
| PTB | RCT | 7 | 1,245 | RR = 0·93 | 0·51, 1·71 | 0·8251 | 20·6% |
|  | Non-RCT | 7 | 4,539 | RR = 0·90 | 0·70, 1·15 | 0·4003 | 7·3% |
| Abbreviations: C-section = Caesarean Section; FPG = Fasting Plasma Glucose; PPG = Post-Prandial Glucose; SGA = Small for Gestational Age; LGA = Large for Gestational Age; GA = Gestational Age; GHTN = Gestational Hypertension; PTB = Pre-term Birth; RR = Relative Risk; MD = Mean Difference; CI = Confidence Interval | | | | | | | |

**Table F3:** Summary of meta-analysis results for maternal outcomes relating to safety comparing remote care to usual care for non-high risk studies

| **Outcome** | **Study Design** | ***k*** | **Observations** | **Pooled effect** | | | **Heterogeneity I^2^** |
| --- | --- | --- | --- | --- | --- | --- | --- |
|  |  |  |  | **Effect Size** | **95% CI** | **p-value** |  |
| Emergency c-section | RCT | 3 | 418 | RR = 0·55 | 0·21, 1·42 | 0·2153 | 65·5% |
|  | Non-RCT | 3 | 6,455 | RR = 0·99 | 0·77, 1·29 | 0·9960 | 9·1% |
| Induction of Labour | RCT | 2 | 419 | RR = 1·04 | 0·62, 1·73 | 0·8934 | 69·2% |
|  | Non-RCT | 7 | 9,669 | RR = 1·04 | 0·89, 1·22 | 0·5919 | 73·8% |
| PROM | RCT | 2 | 418 | RR = 1·95 | 0·49, 7·76 | 0·3456 | 0·0% |
|  | Non-RCT | 0 | - |  |  |  |  |
| Polyhydramnios | RCT | 3 | 538 | RR = 1·08 | 0·74, 1·57 | 0·7006 | 21·6% |
|  | Non-RCT | 1 | 63 | RR = 1·03 | 0·07, 15·79 | 0·9818 | N/A |
| Abbreviations: C-section = Caesarean Section; PROM = Premature Rupture of Membranes; RR = Relative Risk; CI = Confidence Interval | | | | | | | |

**Table F4:** Summary of meta-analysis results for neonatal outcomes relating to safety comparing remote care to usual care for non-high risk studies

| **Outcome** | **Study Design** | ***k*** | **Observations** | **Pooled effect** | | | **Heterogeneity I^2^** |
| --- | --- | --- | --- | --- | --- | --- | --- |
|  |  |  |  | **Effect Size** | **95% CI** | **p-value** |  |
| NICU | RCT | 5 | 788 | RR = 0·74 | 0·49, 1·11 | 0·1408 | 14·9% |
|  | Non-RCT | 7 | 18,197 | RR = 0·97 | 0·88, 1·07 | 0·5690 | 3·2% |
| Jaundice | RCT | 4 | 489 | RR = 1·39 | 0·84, 2·30 | 0·2031 | 0·0% |
|  | Non-RCT | 5 | 3,354 | RR = 0·98 | 0·40, 2·37 | 0·9579 | 77·3% |
| Hypoglycaemia | RCT | 7 | 991 | RR = 1·13 | 0·80, 1·59 | 0·4925 | 0·0% |
|  | Non-RCT | 8 | 8·802 | RR = 0·79 | 0·36, 1·60 | 0·4762 | 88·4% |
| Respiratory Distress | RCT | 3 | 538 | RR = 1·35 | 0·52, 3·50 | 0·5322 | 44·3% |
|  | Non-RCT | 4 | 6,722 | RR = 1·11 | 0·74, 1·68 | 0·6118 | 38·7% |
| Shoulder Dystocia | RCT | 4 | 766 | RR = 0·55 | 0·14, 2·14 | 0·3890 | 22·1% |
|  | Non-RCT | 7 | 9,237 | RR = 0·76 | 0·32, 1·83 | 0·5406 | 64·1% |
| Stillbirth | RCT | 0 | - |  |  |  |  |
|  | Non-RCT | 5 | 16,376 | RR = 0·73 | 0·41, 1·33 | 0·3027 | 0·0% |
| Abbreviations: NICU = Neonatal Intensive Care Unit; RR = Relative Risk; CI = Confidence Interval | | | | | | | |

# Appendix G – Forest Plots

***Effectiveness – Maternal Outcomes – Sensitivity Analysis by Diabetes Type***

**Figure G1:** Forest plot of postpartum HbA1c (%) comparing remote to usual care for GDM non-RCT only studies

**Figure G2:** Forest plot of postpartum 2-hour PPG (mmol/L) comparing remote to usual care for GDM non-RCT only studies

***Effectiveness – Maternal Outcomes – Sensitivity Analysis by Remote Care Type***

**Figure G3:** Forest plot of elective caesarean section comparing remote to usual care for telemedicine in non-RCT studies

**Figure G4:** Forest plot of elective caesarean section comparing remote to usual care for mHealth in non-RCT studies

**Figure G5:** Forest plot of spontaneous vaginal birth comparing remote to usual care for hybrid telemedicine in non-RCT studies

**Figure G6:** Forest plot of operative vaginal birth comparing remote to usual care for telemedicine in non-RCT studies

**Figure G7:** Forest plot of gestational weight gain comparing remote to usual care for mHealth in non-RCT studies

**Figure G8:** Forest plot of hypertensive disorders comparing remote to usual care for web-based systems in non-RCT studies

**Figure G9:** Forest plot of postpartum 2-hour PPG comparing remote to usual care for mHealth in non-RCT studies

***Effectiveness – Maternal Outcomes – Sensitivity Analysis by Risk of Bias***

**Figure G10:** Forest plot of elective caesarean section comparing remote to usual care for low-risk non-RCT studies

**Figure G11:** Forest plot of GWG comparing remote to usual care for low-risk non-RCT studies

**Figure G12:** Forest plot of postpartum 2-hour PPG comparing remote to usual care for low-risk RCT studies

***Effectiveness – Neonatal Outcomes – Sensitivity Analysis by Remote Care Type***

**Figure G13:** Forest plot of macrosomia comparing remote to usual care for mHealth in RCT studies

**Figure G14:** Forest plot of LGA comparing remote to usual care for hybrid telemedicine in non-RCT studies

***Safety – Maternal Outcomes – Sensitivity Analysis by Diabetes Type***

**Figure G15:** Forest plot of emergency caesarean section comparing remote to usual care for GDM only RCT studies

***Safety – Maternal Outcomes – Sensitivity Analysis by Remote Care Type***

**Figure G16:** Forest plot of emergency caesarean section comparing remote to usual care for mHealth in RCT studies

***Safety – Neonatal Outcomes – Sensitivity Analysis by Diabetes Type***

**Figure G17:** Forest plot of NICU admission comparing remote to usual care for GDM only RCT studies

**Figure G18:** Forest plot of respiratory distress comparing remote to usual care for GDM only non-RCT studies

***Safety – Neonatal Outcomes – Sensitivity Analysis by Remote Care Type***

**Figure G19:** Forest plot of NICU admission comparing remote to usual care for mHealth in RCT studies

# Appendix H – Funnel Plots

**Figure H1:** Funnel plots of maternal effectiveness outcomes

**Figure H2:** Funnel plots of neonatal effectiveness outcomes

**Figure H3:** Funnel plots of maternal safety outcomes

**Figure H4:** Funnel plots of neonatal safety outcomes
